# Emergence of network effects and predictability in the judicial system

**DOI:** 10.1038/s41598-021-82430-x

**Published:** 2021-02-02

**Authors:** Enys Mones, Piotr Sapieżyński, Simon Thordal, Henrik Palmer Olsen, Sune Lehmann

**Affiliations:** 1 Technical University of Denmark, DTU Compute, Lyngby, Denmark; 2 Khoury College of Computer Sciences, Northeastern University, Boston, USA; 3 Faculty of Law, University of Copenhagen, Copenhagen, Denmark; 4 Center for Social Data Science, University of Copenhagen, Copenhagen, Denmark

**Keywords:** Applied physics, Computational science

## Abstract

As courts strive to simultaneously remain self-consistent and adapt to new legal challenges, a complex network of of citations between decided cases is established. Using network science methods to analyze the underlying patterns of citations between cases can help us understand the large-scale mechanisms which shape the judicial system. Here, we use the case-to-case citation structure of the Court of Justice of the European Union to examine this question. Using a link-prediction model, we show that over time the complex network of citations evolves in a way which improves our ability to predict new citations. Investigating the factors which enable prediction over time, we find that the content of the case documents plays a decreasing role, whereas both the predictive power and significance of the citation network structure itself show a consistent increase over time. Finally, our analysis enables us to validate existing citations and recommend potential citations for future cases within the court.

As systems of human knowledge grow, networks grow from lists of references which attribute credit to prior work. There are many examples of such networks in the complex systems literature, for example academic citations^1,2^, the world-wide web^3^, and citations between patents^4^. Here, we focus on the network of citations between court cases^5^. These networks are interesting because case law is where abstractly formulated statutory law meets the world of facts, events, and social practices. In this sense, case law is the frontier of law, where it is decided how statutory law should be interpreted. Sometimes case law even supplements the law, when no statutes apply immediately. Citing previous cases is a sign of legal precedent. Legal precedent function as a source of law for the court. By relying on precedent (i.e. it’s decisions in previous cases) the court seeks to uphold consistency in its case law. Identifying what previous cases the court cites in new decisions is a way of grasping what cases the court considers important for the decision of new cases.

In this work we use the fact that the citation graph is a complex network and draw on network science methods^6,7^ to investigate the development of case law through the citation patterns of The Court of Justice of the European Union (CJEU) in order to illuminate underlying factors which shape the Court’s case law ^8,9^.

Specifically, we consider the citations occurring in the period between 1955 and 2014. These form a network of individual cases (nodes) connected by citations (directed links). As time goes by, new cases become part of the network citation structure grows while its complexity increases. In a technical sense, our question is to what extent the (existing) observed structure of the citation network can explain the outgoing citations of new cases. We pose the question as a link prediction problem. Specifically, we define six quantities, or *features*, pertaining to the content of cases and the structure of citations and use them as input variables to predict the existence of each link in the network separately. The prediction is implemented as a recommender system: for a single link, we assign a score to all possible links and determine the rank of the original link in the sorted predictions. Our model provides a measure of the level of predictability of the Court itself.

We begin by providing an overview of the network structure observed in the CJEU, and show that the Court’s citation network develops a non-trivial structure, characteristic of complex networks^6,10^. Based on these observations, we define the six features of the Court’s citations, designed to measure aspects of both content and structure, each feature extracted from case documents or meta-data available in the CJEU database (we provide an example of a case document in the Supplementary Information). We find that the Court’s citations are highly predictable. Moreover, as the Court’s case law develops over time, we find that our predictions become more accurate. Therefore, we investigate the temporal changes in performance and importance of single features. We show that certain properties of cases, for example the similarity between their content or their age, have decreasing significance in describing the observed citations. As a counterpoint, we see an increasing predictive power of features based on the networks structural, such as common citations.

Thus, as we analyze the changes in predictability over time, we are able to form a picture of which mechanisms characterize the Court’s citations by interpreting the importance and the predictive power of the six quantities. In this sense our methodological work enables us to provide new insight into the legal system and its evolution toward greater predictability.

## Results

As the number of references within the court continues to grow, the structure of the citations becomes more complex: we observe a steady increase of the clustering coefficient (defined as the fraction of triangles in the network) after 1980, while the average shortest path between cases (number of references one needs in order to construct a path from one case to another) remains roughly constant over time (in a wide range of simple networks, e.g. randomly connected or regular graphs, the shortest path is expected to grow as the logarithm of the size of the network^11,12^). The network also develops a broad degree distribution, with few very highly cited cases and most cases attracting no or only a few citations. Small values for the shortest path, high clustering, and broad degree distributions are considered hallmarks of complex networks^13–15^ (see Fig. S1 in the Supplementary Information for basic characteristics of the citation network).

The fact that the network develops a complex structure, suggests that neighboring citations might be useful with respect to predicting the citations made as part of cases. The court grows slowly in the early decades (between 1950 and 1980) resulting in fewer than 1000 cases. Furthermore, the CJEU has not established a canonical way to cite prior decisions until the late 1970s’. To minimize the effect of these inconsistencies and ensure the court has a number of citations sufficient to train a recommender system, we start with the network aggregated up to 1978. As we wish to use network structure for the link prediction, we restrict our calculations to the *weakly connected giant component*, that is, we only consider cases that are connected to the largest component of the network (resulting in 8574 cases, $89\%$ of the entire network).

### Link prediction

**Figure 1:**
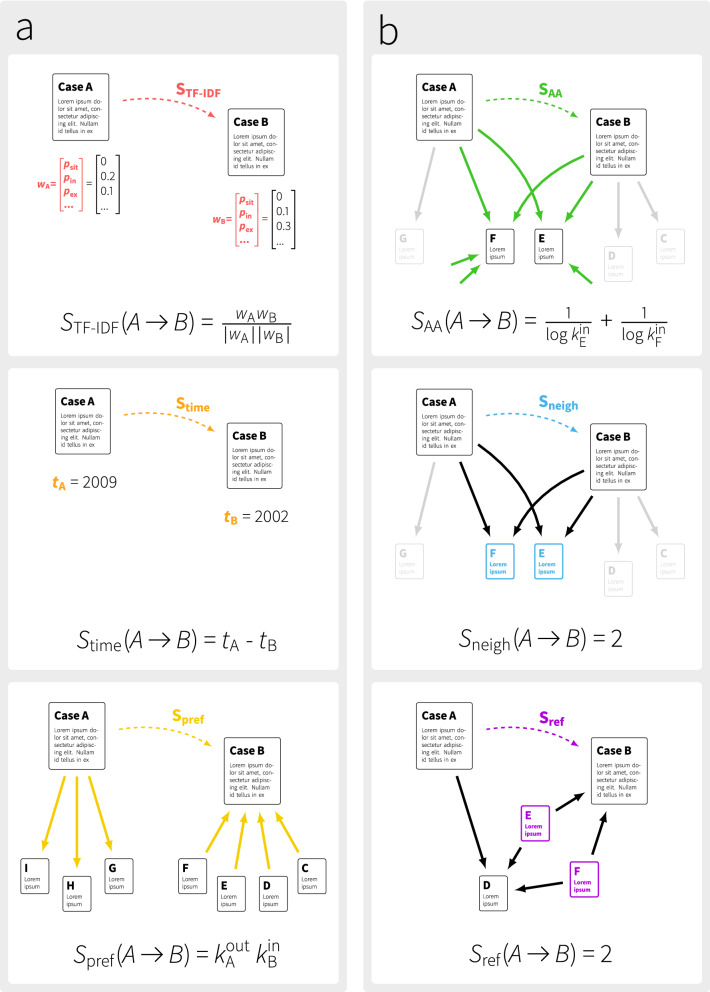
Features used in the inspection of the CJEU court. (**a**) Nodal features: TF-IDF ($S_{\mathrm{TF-IDF}}$) reflects similarity of content, time difference ($S_{\mathrm{time}}$) identifies how contemporary the two cases are; and preferential attachment ($S_{\mathrm{pref}}$) quantifies how many other cases refer to a candidate case. (**b**) Structural features: Adamic-Adar ($S_{\mathrm{AA}}$), common neighbors ($S_{\mathrm{neigh}}$), common referrers ($S_{\mathrm{ref}}$), all of which are inspired by features used in recommender systems and quantify the similarity between network “neighborhoods” of the cases.

We train a Random Forest classifier using six features: TF-IDF (term frequency–inverse document frequency), time difference, preferential attachment, Adamic-Adar, common neighbors, and common referrers (see Fig. 1 for an illustration of each feature). The features can be categorized into *nodal* (pertaining to the content of the cases represented as nodes in the graph, Fig. 1a) and *structural* (Fig. 1b). Full information regarding the features and details of the classifier are provided in the Materials and Methods.

**Figure 2:**
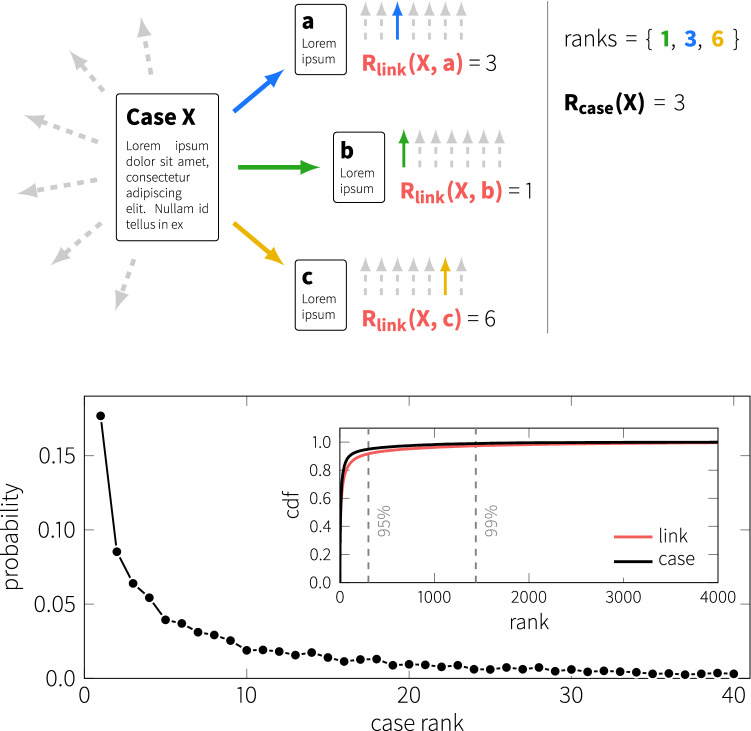
Global performance of the link prediction. Top: definition of the median prediction rank performance measure: each link of a case (colored arrows) is compared against all non-existent links (dashed gray) providing the link ranks (bold red). The full median prediction rank (calculated for each case)is defined as the median rank of outward links corresponding to the case (bold black). Main plot shows the probability mass function of low case ranks, inset shows the cumulative distribution function of the link (red) and case ranks (black). Dashed lines mark the 95% and 99% percentage of the total number of cases.

Our link prediction method is similar to a recommendation system. That is, we retain all but one of the links of a given case and aim to predict the missing link. Further, we evaluate the prediction at the level of individual links: for each link *i*, emanating from node *A*, we remove link *i* and then calculate the score of all non-existent links originating from node *A* (including link *i*) and find the rank of link *i*. This way, we do not evaluate the performance of predicting the original link, but we also obtain a *rank* of that link which characterizes how close is our prediction to the observed real link. We expect existing links to have a higher rank than the majority of non-existing links (see Fig.2).

The aim of our study is to use this machine learning method in order to understand which aspects of the citation network and which nodal properties contain information about the real-world citations. In this sense, we use prediction as a tool to describe each real-world property as a feature in our machine learning algorithm. Hence, we see machine learning as a way to learn about the significance of content and structure; not necessarily as as an actual recommender system to be used in practice. Using our method to recommend possible citations to the court requires careful considerations, as we discuss below.

The general performance of the classifier is quantified in Fig. 2, where we plot the distribution of the *median prediction ranks* over the whole network. The upper panel illustrates how the median prediction rank for a single case is calculated. For each case, we iterate over all links. For each link, we determine the rank of that link when compared against all (non-existent) links emanating from the source case of the link in question, providing the link level ranks. For each case, we determine the median rank measured across its outgoing links. Low rank values imply high performance. The plot in Fig. 2 shows the probability mass function of median ranks. Prediction of the links is surprisingly efficient, and the probability in Fig. 2 (lower panel) drops exponentially beyond low values (for degrees above 5). This effect is even more clearly shown in the cumulative distribution (cdf): 95% of the cases have a median rank below 292 and 99% of the cases exhibit ranks below 1335. Ranks of this magnitude are surprisingly low considering that the vast majority of links are ranked against thousands of non-existent links, suggesting that references in the court are highly predictable even using this small set of simple features.

### How the model identifies individual cases

In order to better understand how the random forest algorithm identifies which cases to cite, we now describe the underlying mechanics of the classification in detail. In our case, the goal of the algorithm is to decide if a link exists or not. The random forest classifier is an ensemble of individual decision trees trained slightly differently.

A single decision tree is a binary structure where each node embodies a binary decision based on the value of a single feature. These binary decisions are, in most cases, comparisons against a reference value that is adjusted during the algorithm’s training phase. For instance, a node may represent the question “Does this case have at least 4 citations in common with the index case?”. If the answer is ‘yes’, the case is sent down one branch, if not it is sent along the other branch. In this way, the prediction process is a sequence of such consecutive comparative binary questions. The bottom most nodes (the *leaves*) of the tree then assign a class to the case at hand: is it a possible reference or not?

Individual trees are prone to various problems, most importantly over-fitting. Over-fitting can be avoided a by using a so-called ‘random forest’ approach, where we introduce many decision trees that are grown in a stochastic way, e.g., by using a subset of data points and limiting trees to rely on only a subset of features. Once all training data are considered and all trees have been fitted, the score assigned to a specific case is based on the fraction of trees that assigned the link as a real reference.

To understand the algorithm’s decision-pipeline, we study how often the trees use a feature at different stages of the prediction. More precisely, we ask how frequently a feature is used in the different levels of the decision trees. Comparison of the features and our main results are discussed in details in the Supplementary Information. This exercise is interesting because the features that tend to be used early in the tree (near the root) have larger discriminatory power; these features allow the algorithm to label the largest possible fraction of cases as *not relevant*. Features used late in the tree (near the leaves), help to refine the decision, separating the right case from others that are similar to it.

**Figure 3:**
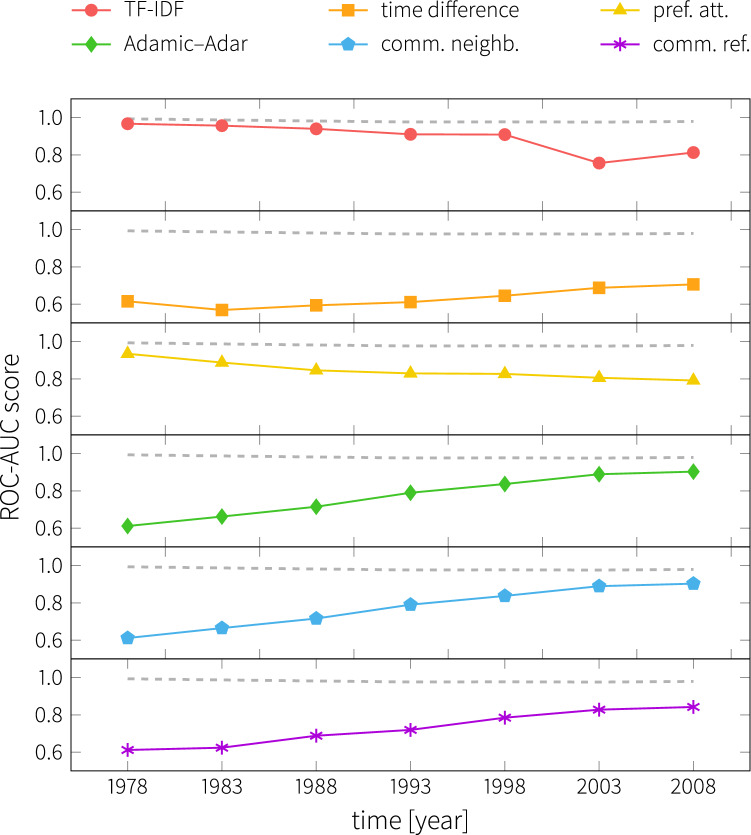
Details of feature usage inside the decision trees. The curves show the fraction of decision nodes in the decision trees that use a specific feature in different levels of the trees (they add up to one). For each feature, we calculate the number of (internal) decision nodes that make the split based on the value of that feature, normalized by the total number of nodes in that level. Results are averaged over all trees in a random forest and over 5 independent forests. Black lines indicate the boundary between nodal (lower) and structural (upper) features.

**Figure 4:**
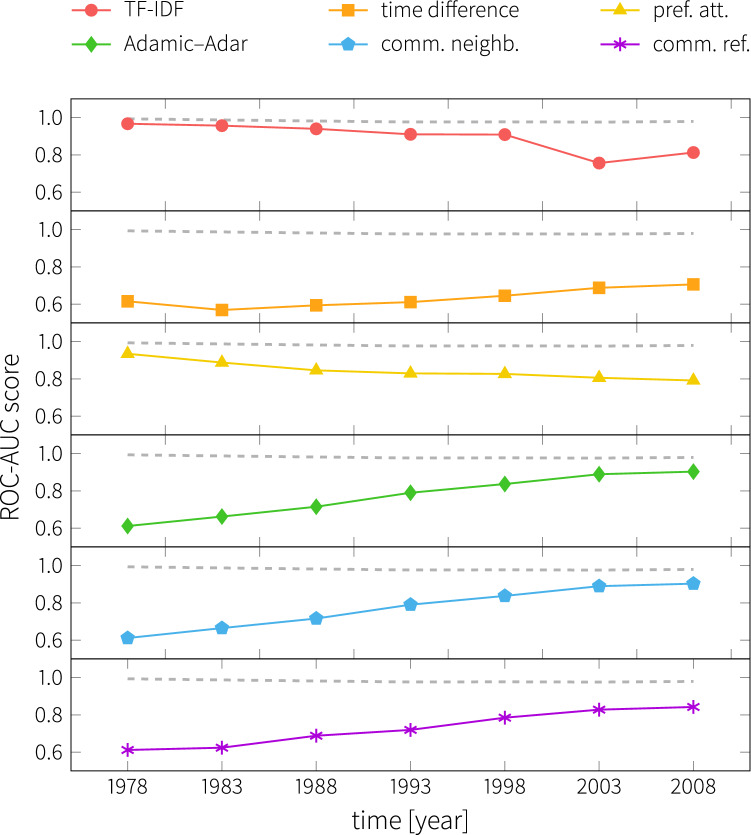
Predictive power of individual features. Lines show the change in ROC-AUC for each feature calculated from the raw feature values. The dashed gray lines show the value 1.0 as a guide to the eye.

As a measure of which level in the trees a feature is typically used, we compute the relative frequency of features aggregated over the entire forest. For a feature *f* and a level *l* of the trees, we define $p_f(l)$ as:

1 $$\begin{aligned} p_f(l) = \frac{\sum _t n^f_t(l)}{\sum _t n_t(l)}, \end{aligned}$$

where $n^f_t(l)$ is the number of split (decision) nodes using feature *f* in level *l* of tree *t*, and $n_t(l)$ is the width level *l* of tree *t* (total number of nodes in that level). The values of $p_f(l)$ are averaged over five different realizations of a random forest and shown in Fig. 3. Each line in the figure represents results for the citation network aggregated up to a specific year, corresponding to the same years as in Fig. 4. Black lines in the stacked histograms distinguish between nodal (lower) and structural (upper) features and indicate the overall trend of feature usage between the two categories.

Overall it is clear that nodal features (TF-IDF, time difference, and preferential attachment) and structural features (Adamic–Adar, common neighbors, and common referrals) are used differently by the algorithm over time. In the most recent network (incorporating all data), classification at the root level is based solely on structural features; the large splits of data are based on network structure. As we move closer to the leaf nodes, refining the decision among groups of similar cases, the nodal features dominate the decisions. As we move backwards in time, incorporating less and less data, this trend is less strong and nodal features (especially the textual similarity encoded through the TF-IDF feature) play a significant role.

This means that when identifying the references in the full network data, the classifier treats nodal and structural features in a fundamentally different manner than in the early court. First the algorithm uses network structure to finds the right network neighborhood. Then nodal features are used for fine-tuning the decision.

### Evolution of feature importances

Having discussed the recommendation mechanism in detail, we are now able to use these methodological considerations to analyze the mechanisms that are at play in the court. To do so, we study how the feature importances change over time. Studying how the model identifies individual cases pointed us to interesting patterns in predictability over time as the network of cases grows. In this section we analyze the performance of the model by explicitly assessing the predictability of incoming citations as a function of time. Consistent with our analysis of the random forest, we find that the importance of features changes as the citation network grows in size and becomes increasingly complex over time. As we show in 'Network growth' in Supplementary Information, these changes are not a trivial consequence of the network growth, but instead they characterize a particular behavior of the Court over time.

To understand which mechanisms most influence the observed performance, we first analyze the features individually. Specifically, we calculate ROC-AUC for each feature alone, using the raw feature values (see Materials and Methods for details). Fig. 4 shows time evolution of ROC-AUC of the classifiers by each individual feature, based on 5-year periods. The nodal features, shown in the top three panels of Fig. 4 (TF-IDF, time difference and preferential attachment) show mostly decreasing trends, with only time difference indicating a slight increase. However, the corresponding value of ROC-AUC for time difference is close to that of a random classifier (an ROC-AUC of 0.5). All of the structural properties, shown in the bottom three panels of the figure, display a significant increase of predictive power. This development explicitly shows that the case-to-case network structure allows us to infer the links with growing accuracy and precision. Further analysis with point-biserial correlation^16^ confirms these observations: nodal features show limited and vanishing correlation, whereas structural features exhibit a steady improvement in terms of performance. Note that in case of time difference, we used the negative of the feature to obtain a positive correlation between the feature and the predicted observable. Beyond feature ROC-AUC and correlation, we investigated the predictive power of features by training a classifier using a single feature and then measuring its performance; results using this method remain consistent. Furthermore, to assess the extent to which much the model draws on each feature, we also measure feature importance and its change over time. Detailed analyses on the features support the above observation (see Fig. S8 of the Supplementary Information for details).

Both the changes in predictive power and development of feature importance suggest that the relative usefulness of the content of individual cases, i.e., the nodal characteristics, decreases over time. At the same time, we observe the emergence of complex network structure among the court’s judgments, allowing for more accurate predictions. A possible explanation of these observations is that the content of the documented cases does not change significantly over time: there are strict rules of the content generation when a case is represented in the database. However, this is not the case with the network structure as this structure is not controlled by any regulation, it is only affected by the citation culture developed within the court. In this sense, the network structure of citations is an emergent property of the court. Our results show that this network is becoming increasingly informative of the actual references.

### Interpreting model errors

Continuing the analysis of the legal system through the lens of link prediction, we note that from a legal perspective, it is interesting to study the situations where the model makes mistakes. Here we focus on false positives and false negatives. In the case of false positives, our model recommends a reference between two cases, which in turn does not exist in the citation network of the court. Empirically, these are cases that, according to the algorithm, are ‘supposed’ to be cited. These cases discuss similar legal topics, but with subtle differences in the specific details of the legal issues (see Supplementary Information for details). An illustrative example is the suggested citation of Case C-412/05 P in Case C-304/06 P. Both of these cases deal with Community Trade Mark Law, including the distinctive characteristics of the mark. However, the suggested citation is concerned with the procedural issue of appeal (in a trademark law case) whereas the citing case in concerned with substantive trademark law. It is this difference in the particular focus of the cases which the algorithm was not able to discover.

On the other hand, false negatives are the references that were not found by our model but are observed in the court. There are several reasons for false negatives. First, it is common to cite previous cases to provide an example for a type of argument even though the example itself is not about the same legal topic in which the argument is used. A legal principle which is used across many different legal topics then leads our algorithm to generate false negatives. An example is Case 124/83 citing joined cases 94-63 and 96-63. The citation emerged as evidence of a general principle that an authority which adopts measures affecting the persons concerned or which withdrawn a favorable decision must bear the burden of proof itself. An interesting avenue for future work would be to employ more sophisticated natural language processing methods to detect situations where similar legal principles are at play. Second, false negatives also include clerical errors—the citations are mistakes as another case should have been cited. For more details, see Tables S1–S3. in the Supplementary Information.

## Discussion

In this paper, we have shown that network science methods and machine learning techniques can be useful tools for understanding the patterns of how case law is applied in a rapidly growing corpus of of legal decisions.

### Importance of understanding empirical patterns of case law usage

A deeper understanding of the principles that shape the application of statutory law is key, since consistency in how cases are treated, not only supports equality before the law, but also enhances predictability and effectiveness. Predictability is desirable because when those who are subject to the law know that new cases will be treated consistently with previous cases, they can use those earlier cases as a legal compass, to navigate their behavior in accordance with the law. Currently, maintaining consistency and predictability is expensive. It requires an insight into and overview of previous case law, which is increasingly difficult for a single human being to achieve.

Moreover, while many cases have little general relevance (e.g. regarding uncommon scenarios, or trivial repeat cases) a few key cases have proven cardinal in understanding unwritten legal principles and explaining statutory law, and others are important for very specific situations^17^. Important cases are currently identified by scholars and lawyers simply reading court cases. We posit that it may be helpful to introduce tools for information management based on methods such as the ones proposed here in order to help individuals to navigate the case law. Case law databases make it possible to index cases by specific categories, but the cases in the database must first be categorized in a way that supports legal reasoning. Improvements in search engines have made it possible to do full text search, making the job of finding applicable case law considerably easier. However, knowing what to look for, given a particular problem, remains a skill reserved for legal professionals and is susceptible to these professionals’ own biases and other human limitations^18^.

### Applications

This paper shows that there is a possibility of predicting which cases are applicable as precedent given the content (text and citations) of an already existing case. While it has been argued that computerized recommender systems cannot supplant ‘lawyer’s craft’^18^, we argue that algorithmically identifying relevant cases may have several advantages, for example improving the reproducibility of doctrinal legal studies and reducing individual bias. Introducing a technology that is capable of interacting with the insight of expert humans has potential to bring several advantages to the legal sector overall. In the following we list the most obvious applications of our link prediction system (see SI for full discussion). *First*, transferring information from CJEU to domestic settings. E.g. making it easier for administrative agencies to make informed decisions about rights of citizens. *Second*, supporting legal service providers in finding relevant CJEU case law to support arguments made for clients. *Third*, support to the CJEU itself. A link prediction system could help the court navigate its own case law when preparing new cases and may even be used to check whether a new case decided by the court sufficiently cites relevant former cases. *Finally*, the link prediction system could be implemented in legal research and teaching settings. Allowing students and legal scholars to navigate case law in more advanced ways could potentially allow for new insights by legal scholars and law students.

### Risks and limitations

One of the proposed applications of our findings would be to translate them into a recommender system for use in legal practice. Before doing so, one must consider the risk of adverse effects. Here, we highlight what we consider to be the most important issues: automation bias, the cold start problem, and citation specificity.

Automation bias is the tendency towards favoring machine-generated suggestions or decisions, often despite opposing information that did not come from an automated system ^19^. This bias exists in at least three versions: commission (relying on wrong information), omission (relying on incomplete information) and complacency (insufficient attention to and monitoring of automation output). Introducing an automated recommender system to the process of legal research is likely to also bring about automation bias in legal behavior, especially in the form of omission and complacency. Omitting relevant precedent as a result of automation bias is doubly problematic. First, the omitted precedent might turn out to be more relevant to the specific case at hand than those recommended by the system. Second, relying only on recommended cases will produce a feedback loop to the recommender system that will further increase the weight of the precedents being recommended (if, as we presume, the new decision will be fed into the network that is used for recommendation). In order to overcome the problems associated with automation bias, we would suggest that a recommender system be modified in ways that can counteract the issues described above. It must provide enough variation in presented cases and their ordering in an attempt to mitigate complacency ^20–22^.

Recommendation systems also suffer what is known as the ‘cold start problem’, where a new piece of content (here: a new case) does not have enough information associated with it, since it has not yet been used (here: cited). A recommender system could therefore stifle jurisprudential development by not recommending newer cases. Although several features of our model (TF-IDF, common neighbors, Adamic-Adar), do not rely on existing citations of a decision to start recommending that decision, we still believe there is a need to consider and counteract the cold start problem. We suggest that new cases must be given more visibility in the recommender system. This can be done by backward linking: the previous cases cited by a new case, may be used as an indication of the relevance of the new case in the context of what those cited cases represent in the network. The recommender system could also be constructed in a way that it assigns more weight to recent cases than to old cases. It could also be built in such a way that it always shows the most recent cases of a similar kind along with those cases that carry the best predictive values for a given situation.

Furthermore, we note that in law, one does not refer to entire cases but to the specific part (paragraph) of a case which is relevant to one’s argument^23^. In this sense our recommendations—which refer to entire cases—are not specific enough. We expect that our methods that rely on citation structure and the content comparison can be extended to recommend case paragraphs rather than entire cases, however due to non-uniform paragraph labeling in the dataset, the transition to paragraphs falls outside of the scope of this article.

Finally, it should be noted that what we observe in this paper is the fact of citations existing from one case to another (previous) cases. These citations occur in the text of the published case documents as found in the publicly available EUR-LEX database. It is generally accepted that courts rely on their own previous case law when they decide new cases. How those case are reflected in the reasoning of the individual judges that participate in making the decision is, however, hidden from view. All we can access are the exterior signs of that reasoning as found in the published text of the decided cases. Since these decision texts are generally relied upon by lawyers when making legal arguments in new cases, we assume these texts to be the most objective and representative source in regard to the legal reasoning underlying the case decisions. From this assumption follows that we take the fact of citation to reflect the role of the cited case in the reasoning of the citing case.

### Conclusion

In this paper, we have investigated the predictability of citations in the CJEU. Using a recommender system based on three node-related features and three network dependent system, we offer a number of findings. *First*, the court’s citations are highly predictable, with predictability increasing over time. *Second*, we developed an analysis of the model to let us understand how it reaches decisions. Based on this analysis, we found that the factors which enable us to predict change over time, with network features gaining importance as we get closer to the present. *Third*, when we investigated errors made by our model, we discussed how these errors can help us both ‘debug’ the court itself by identifying omissions and clerical errors, as well as the algorithm, highlighting subtleties and nuances not incorporated in the current features. *Finally*, we discussed the ways in which our findings are likely to impact how courts function in the future, across a number of dimensions. We also discussed potential problems associated with using automated citations in the legal process, analyzing the key issues of cold-start and automation bias.

### Model

We used a Random Forest classifier in the link prediction task due to its ability to capture non-linear relationships and it also provides a built-in means for measuring the importance of different features^24^. Here, we use link prediction to inspect two different aspects of the court: predictability and the importance of the features. We calculated six features that can be categorized as nodal and structural properties of the cases. Here we give a short summary of the definition of features and the motivation behind each.

*TF-IDF*—TF-IDF is used to estimate the similarity of two cases based on whether they use the same terminology. This feature first builds a vector of words in each case document, and then calculates the cosine similarity between two judgments^25,26^. This feature assumes a high value when two cases are similar w.r.t their content. Mechanism: quantifies the tendency to cite cases that are relevant to the legal field of the current case.

*Time difference*—is the difference in the years between the cases. Mechanism: encodes the tendency to cite recent, up-to-date rulings.

*Preferential attachment*—This feature is based on the phenomenon observed in many human-made networks that grow over time: as the network evolves in time, nodes having a large number of links tend to collect links more rapidly than those having a few links. This is due to the underlying preferential attaching mechanism that connects new nodes to existing ones with a probability proportional to their number of links. The corresponding feature is calculated simply as the product of the degrees:

2 $$\begin{aligned} S_{\mathrm{pref}}(A\rightarrow B) = k^{\mathrm{out}}_{\mathrm{A}} k^{\mathrm{in}}_{\mathrm{B}}, \end{aligned}$$

where *k* is the number of inward/outward citations of the node. We consider $S_{\mathrm{pref}}$ as a nodal feature, since the driving mechanism that enables judges referring to cases that are already highly cited is rather a social phenomena (and how information spreads in the community of the judges) than a strongly structural one. Mechanism: highly cited cases tend to have high visibility that attracts further citations, and the longer a reference list is, the more likely to cite any other specific case.

*Common neighbors*—The number of other cases both cases cite. Mechanism: the tendency to cite a case that has much in common (that cites the same set of other cases).

*Adamic–Adar*—This feature was developed by Adamic and Adar in^27^, to mine relationships on the web and has since been re-purposed by several studies as a general tool for predicting links. The feature value for a citation from case *A* to case *B* is

3 $$\begin{aligned} S_{\mathrm{AA}}(A \rightarrow B) = \sum _{c \in \Gamma (A) \cap \Gamma (B)} \frac{1}{\log |\Gamma (c)|}, \end{aligned}$$

where $\Gamma (\cdot )$ denotes all citations (inward/outward) of the case, that is, the set of cases it is citing/cited by. Mechanism: similar to common neighbors, but corrects for the bias caused by highly cited hubs, that is, a commonly cited case with few incoming citations is more valuable than a hub following the intuition that it represents a more unique relationship between the two cases citing it.

*Common referrers*—The common referrers feature is an extension of the common neighbors which assumes if case *A* shares some citations with case *c*, then the remaining citations of *c* are also good candidates to cite by case *A*. That is, with high overlap of citations can lend potential citations from each other. In mathematical terms, it is formulated as the overlap between the outward citations of case *A* and the inward citations of case *B*:

4 $$\begin{aligned} S_{\mathrm{ref}}(A \rightarrow B) = \big | \{\Gamma _{\mathrm{in}}(B) \cap \Gamma _{\mathrm{in}}(c) \, \forall c \in \Gamma _{\mathrm{out}}(A)\} \big |. \end{aligned}$$

Mechanism: the citees of other rulings that refer to the same cases are potential candidates for citations.

### Predictability

When predicting a single link of a specific judgment, a prediction trial assigns a score to all links defined by the probability to be a real link, and then ranks them according to their score. Each link is predicted separately, using information available from the rest of the links, that is, we keep all links but the one we predict and then perform the calculations with the random forest. The predictability of a case is defined as the median rank of its true links when all of its connections are probed. If a case is highly predictable, we expect its links to appear at the top of all the ranked links resulting in a low median rank. For a community, we simply define its predictability by the median predictability of its member cases. To measure the predictability of the entire network, we also calculate the ROC-AUC (area under the receiver-operator curve), as it is insensitive to class imbalance and due to its intuitive interpretation: it is the probability that a randomly selected existing link is ranked better than a non-existing link. Alternatively, ROC-AUC is shows explicitly how much better the classifier performs compared to a random guess.

## Supplementary Information

Supplementary Information.
