# Author Correction: Emergence of network effects and predictability in the judicial system

**DOI:** 10.1038/s41598-021-91556-x

**Published:** 2021-06-01

**Authors:** Enys Mones, Piotr Sapieżyński, Simon Thordal, Henrik Palmer Olsen, Sune Lehmann

**Affiliations:** 1 Technical University of Denmark, DTU Compute, Lyngby, Denmark; 2 Khoury College of Computer Sciences, Northeastern University, Boston, USA; 3 Faculty of Law, University of Copenhagen, Copenhagen, Denmark; 4 Center for Social Data Science, University of Copenhagen, Copenhagen, Denmark

Correction to: *Scientific Reports* 10.1038/s41598-021-82430-x, published online 02 February 2021

This Article contains an error, where Figure 3 is a duplication of Figure 4. The correct Figure 3 appears below as Figure 1.

**Figure 1:**
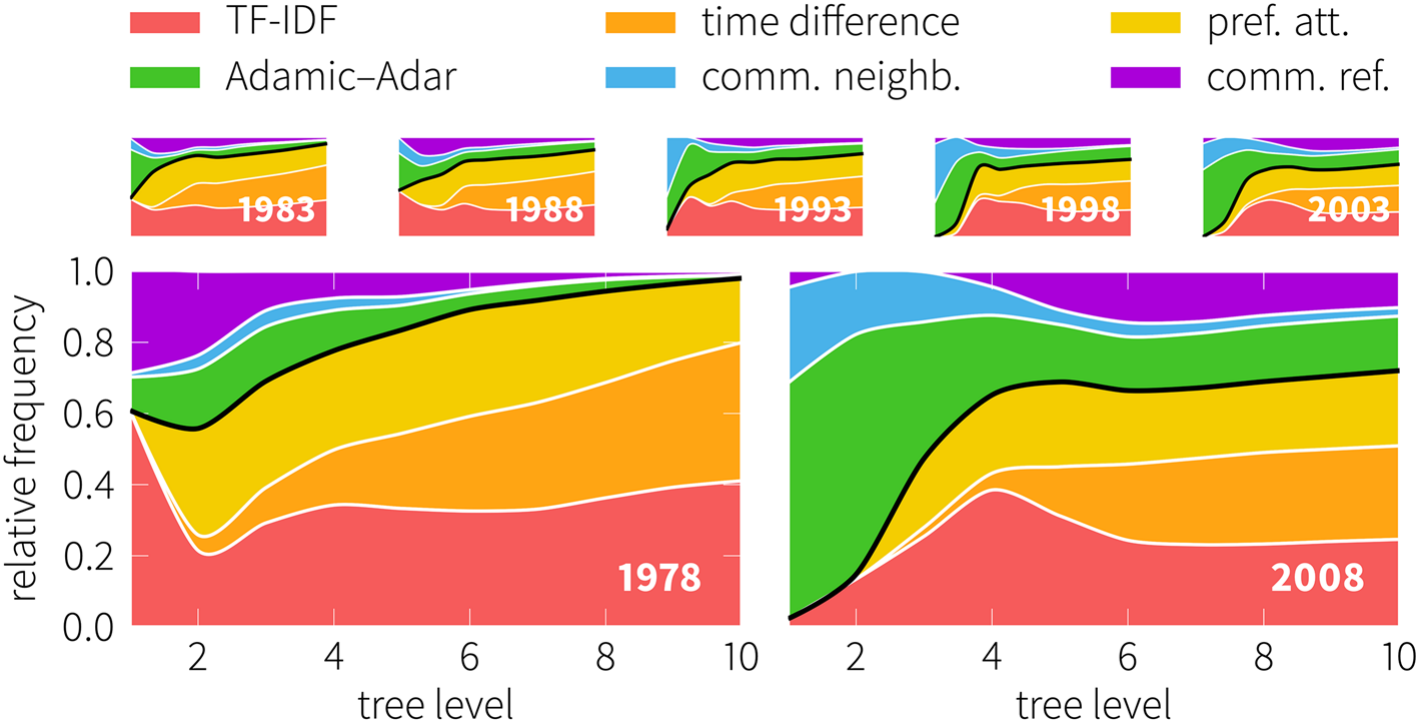
A correct version of the original Figure 3.

