# Supplementary material for: Emergence of network effects and predictability in the judicial system
Source: Sci Rep. 2021 Feb 2;11:2740. doi: 10.1038/s41598-021-82430-x (PMC7854739; doi:10.1038/s41598-021-82430-x)
Supplement: Supplementary file 1 — Supplementary Information. [file 41598_2021_82430_MOESM1_ESM.pdf]

# Emergence of network effects and predictability in the judicial system Supplementary Information

Enys Mones[1] Piotr Sapieżyński[2] Simon Thordal[1,3]
Henrik Palmer Olsen[4] Sune Lehmann[1,5]

[1]Technical University of Denmark, DTU Compute
[2]Khoury College of Computer Sciences, Northeastern University
[3]Ulobby, [4]University of Copenhagen, Faculty of Law
[5]University of Copenhagen, Center for Social Data Science

## 1 Data

Our primary data set consists of the documented cases of The Court of Justice of the European Union (CJEU), which have been collected as XML documents from https://hudoc.echr.coe.int. The XML documents contain information about each case: citations to other cases, starting and ending dates, and the transcript of the case. For illustration purposes, below we provide an excerpt from the transcript of case C-304/06 P with citations to previous cases marked in bold.

> 54. It must, first, be borne in mind that, while the Court of Justice has had occasion to find a degree of overlap between the respective scope of the absolute grounds for refusal to register a trade mark set out in Article 7(1)(b) to (d) of Regulation No 40/94 (see, by analogy, as regards the identical provisions of Article 3(1) of First Council Directive 89/104/EEC of 21 December 1988 to approximate the laws of the Member States relating to trade marks (OJ 1989 L 40, p. 1), Case **C-363/99** Koninklijke KPN Nederland [2004] ECR I-1619, paragraph 67, and Case **C-265/00** Campina Melkunie [2004] ECR I-1699, paragraph 18),

> it is nevertheless the case that, according to established case-law, each of the grounds for refusal to register listed in Article 7(1) of Regulation No 40/94 is independent of the others and requires separate examination (see Joined Cases **C-456/01 P** and **C-457/01 P** Henkel v OHIM [2004] ECR I-5089, paragraph 45; Case **C-64/02 P** OHIM v Erpo Möbelwerk [2004] ECR I-10031, paragraph 39; and Case **C-173/04 P** Deutsche SiSi-Werke v OHIM [2006] ECR I-551, paragraph 59).

We constructed two data sets of different kind: first, a set that includes all content-related information (recordings of the case, starting date) and is attached to each case. Second, a citations network which is constructed from the references between cases: nodes are the cases, and references constitute the links. Whenever there is a case ($A$) that cites another one ($B$), there is a directed link ($A \to B$) in the network. Before any of the analyses is carried out, we perform several filtering steps (described below) to minimize noise.

## 1.1 Content data

To extract information about the content using TF-IDF vectors, we first convert each document to a bag-of-words based on its paragraphs. That is, we remove titles, meta data, list of judges, section titles from the documents and construct the TF-IDF vectors from the content of all paragraphs. These include facts, references to related law, reasoning and the decision. Note that case IDs (and therefore references) are ignored when TF-IDF vectors are constructed.

## 1.2 Network data

We perform our analysis only on cases that are part of the network's giant weakly connected component, omitting the isolated cases. This component is the connected subgraph of the citation network that contains the largest number of cases irrespective of the direction of citation (and time in this case). The giant component consists of 8 574 cases and 43 595 references. The importance of the isolated components is negligible for link prediction as such components contain fewer than 0.5% of all links. As a first overview of the network structure, we plot the distribution of inward and outward citations in the static network (disregarding time) in Fig. S1. Around 18% of

the cases are not cited and 13% of them do not have any references to other cases over the observation period.

The court grows slowly in the early decades (between 1950 and 1980) resulting in fewer than 1000 cases. Furthermore, the CJEU has not established a canonical way to cite prior decisions until the late 1970s'. To minimize the effect of these inconsistencies and ensure the court has a number of citations sufficient to train a recommender system, we start with the network aggregated up to 1978.

An overview of the citation network is shown in Fig. S2, which shows the temporal trend of various network metrics. The network grows exponentially as shown by the number of cases, and after a transient period of 25 years, the average degree also starts to increase monotonously. The average shortest path (measured in the giant connected component) shows a rapid jump after 1978, the same year when the average degree begins to increase, and the paths within the court starts to shrink after the mid 80's. We also report the undirected clustering coefficient, which is the fraction of triangles a case participates in, compared to all possible triangles given its number of inward/outward citations. We considered the undirected clustering coefficient for two reasons: first, the direction of a triangle is not informative in describing the local density of the network (and leads to lower triangle density). Second, directed triangles describe the motif pattern of a network, which, although can be characteristic of the network function and behavior, is not within the scope of this study. As for the undirected clustering coefficient, we observe a slight increase after the first few years. The increasing clustering with the shrinking shortest path is a sign of emergent complexity in the court. As we show in section Context and Structure, the court grows in a non-trivial fashion, resulting in a globally sparse but locally dense network.

# 2 Model

## 2.1 Features

In the current analysis, we explore how the complex interplay between mechanisms based on meta-data and the global structure of the citations shapes the evolution of the court. We apply a simple classifier to predict the citations and through the detailed examination of the model itself, we can learn about

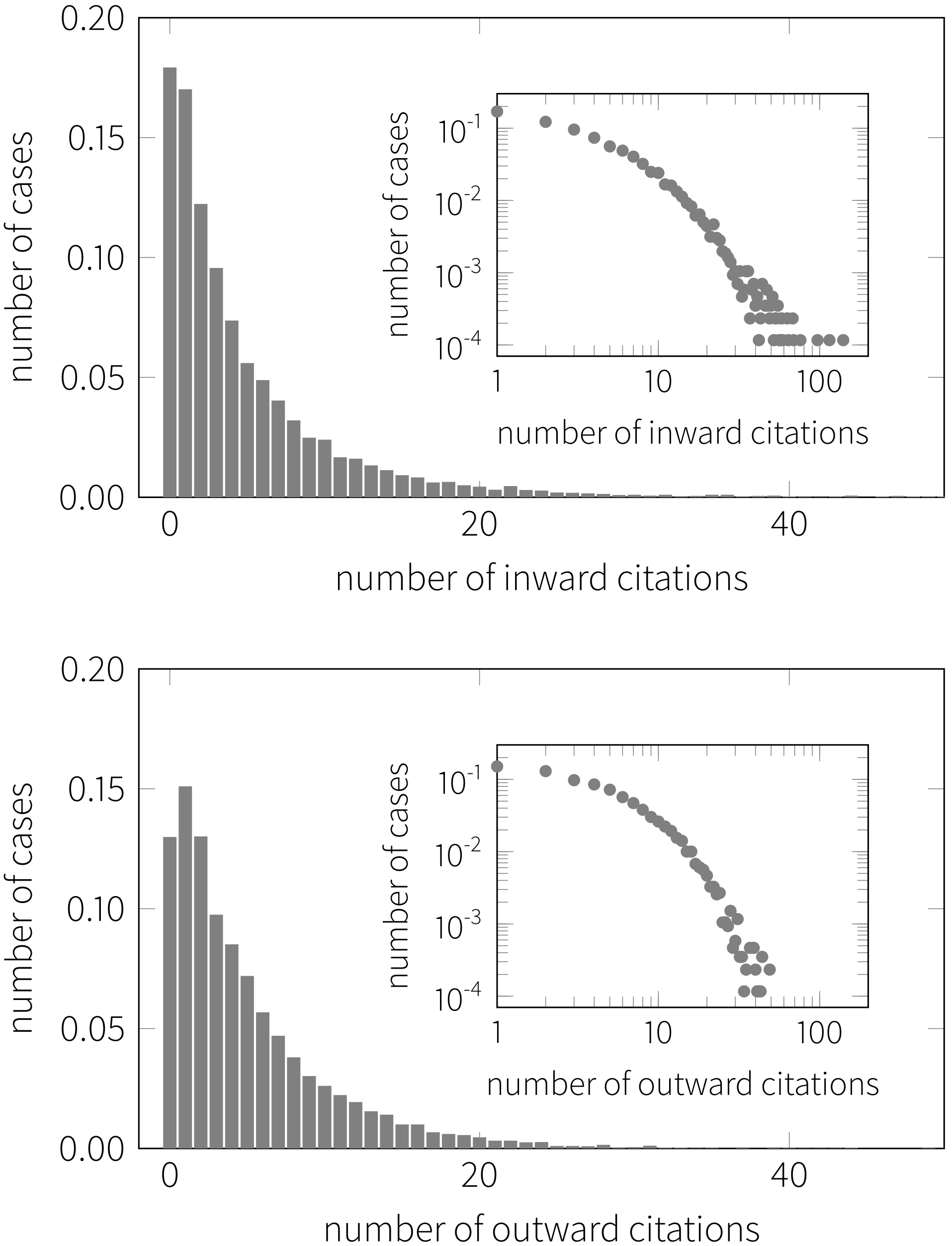


Figure S1: **Distribution of inward- and outward citations in the giant weakly connected component**. The relative frequency of inward citations (top) and outward citations (bottom) in the CJEU. Insets show the same distributions in log-log scale.

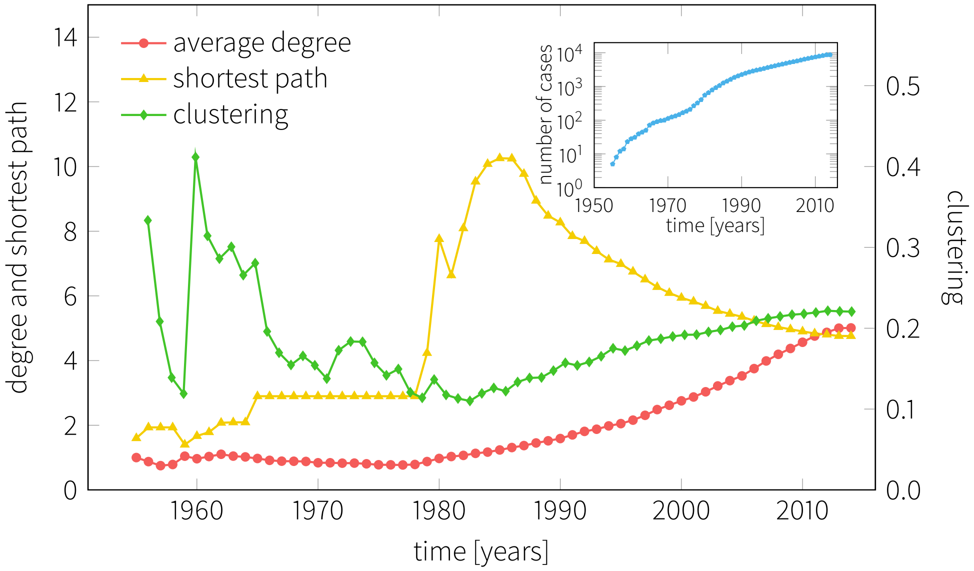


Figure S2: **Evolution of structural properties in the CJEU citation network**. Shown are the average degree (red circle), shortest path (yellow triangle) and clustering coefficient (green diamond) of the network. Axis label for the clustering coefficient is on the right side of the plot. Inset shows the number of cases in the same period.

the court. To this end, we defined six features that correspond to the content and the structure of the citation network. The content of the documented cases is described using the following features: TF-IDF cosine similarity between cases ($S_{\mathrm{TF-IDF}}$), time difference of the decisions in days ($S_{\mathrm{time}}$), and the product of their respective citation counts ($S_{\mathrm{pref}}$). This category contains features that are connected to the documents, or strongly related to the legal community itself. Therefore, preferential attachment is categorized as content-related, since the number of citations a case received strongly influences the extent to which the decision makers are exposed to the case. The three structural features are the Adamic–Adar similarity ($S_{\mathrm{AA}}$), number of common neighbors ($S_{\mathrm{neigh}}$), and the common referrers ($S_{\mathrm{ref}}$). $S_{\mathrm{ref}}$ is similar to $S_{\mathrm{neigh}}$ but it includes 2-hop neighborhood in the network. All features are defined for pairs of cases, and their value depends on the relationship between these pairs of cases.

The motivation behind this particular choice of features is to distinguish between the content and structural development of the court, rather than maximize the prediction performance. That said, although all features grasp a distinct, well defined aspect of the court, they are not fully independent. Figure S3 displays the correlation between the six features. Time difference is the most independent of all and common referrers has the highest correlation in overall. As expected, Adamic–Adar strongly correlates with common neighbors. However, the inclusion of both metrics is explained by the fact that they correspond to slightly different aspects. Common neighbors can be high as a result of overlap in the committee of a decision as well as takes topical similarity into account. On the other hand, Adamic–Adar corrects for the inherent biases present in the system caused by the over-representation of cases that became famous in the community by normalizing by the degree of the common neighbors.

A general overview of the features is shown in Fig. S4, where we plot the distribution of the feature values measured on the links, compared to the distribution over all pairs of cases. Note that the figure serves merely as a comparison of the feature value on the links and between all possible pairs. As expected, the links represent case pairs with higher feature values, as seen in the wider distribution of most of the features over the links. The widening of the distributions indicates a strong differentiating power in case of TF-IDF, preferential attachment, Adamic–Adar, common neighbors and common referrals. However, time difference displays a narrow distribution, meaning that most of the cases actually cite recent cases. The distribution

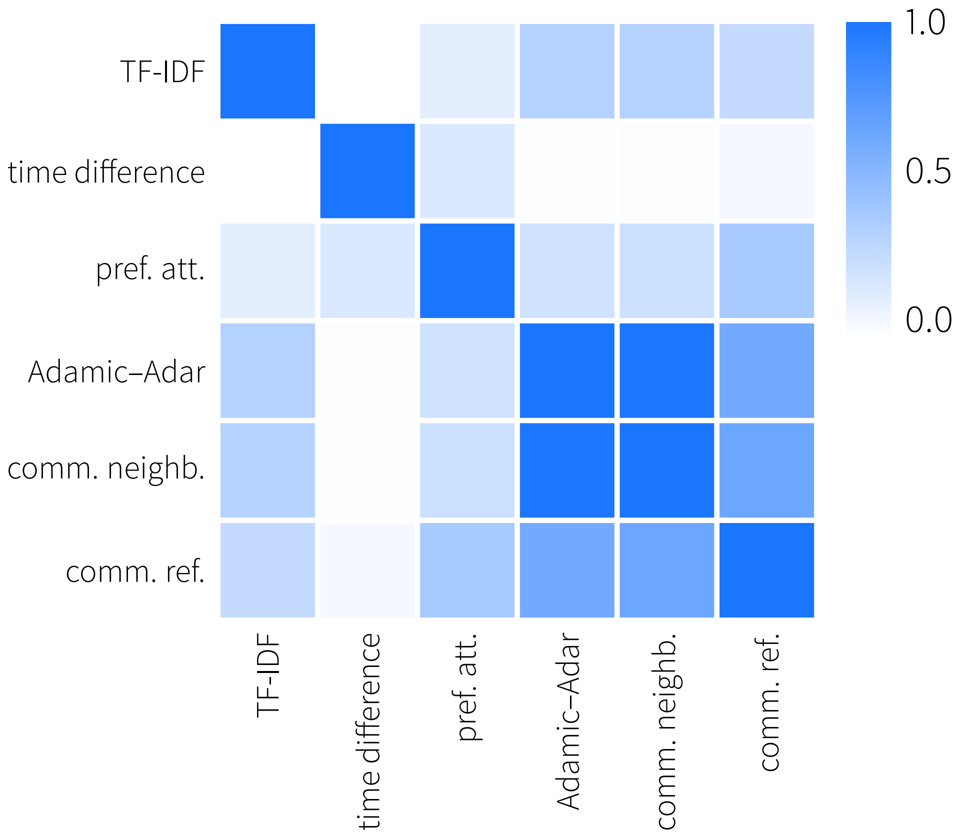


Figure S3: **Correlation between features**. Color of the tiles show the Spearman-correlation between each pair of features.

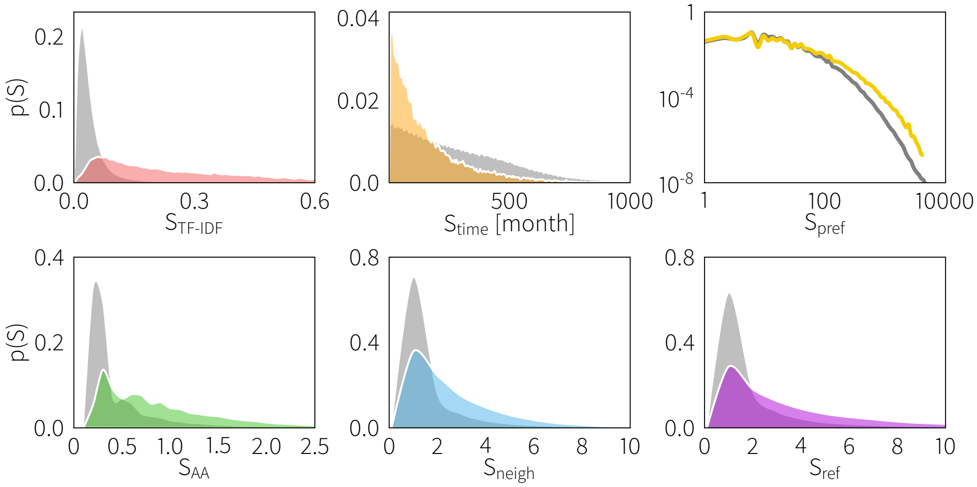


Figure S4: **Distribution of feature values**. Colored curves and areas show the histogram of features over all pairs of cases that lie on the same link. The gray counterparts of the figures correspond to the distribution over all possible cases, irrespective if they are connected or not. For each feature, we applied a different binning size.

almost vanishes around 300 months (25 years), marked by a clear cutoff for the court. Cases have strongly varying length, age, citations, etc., all of which can affect the feature values they are likely to contribute to. In order to see if these meta properties of the documents affect the resulting features, for each case we average out the feature values over their references (outward citations). Results are shown in Fig. S5, with correlations between all features and document length (measured by word count in the paragraphs), age of the case (in years), duration of the case (in months) and references (outward citations). The last column shows the Spearman correlation for the features for comparison. The first observation is that duration of the decision does not correlate with any of the features. Most importantly, with the exception of age, $S_{\mathrm{TF-IDF}}$ shows the lowest correlation in all cases, meaning that the length of the document does not affect the results obtained for $S_{\mathrm{TF-IDF}}$ significantly. The highest feature correlation is observed with the references of the case. This is a result of the averaging method: as feature values have large fluctuations from link to link, a single case has strongly varying features

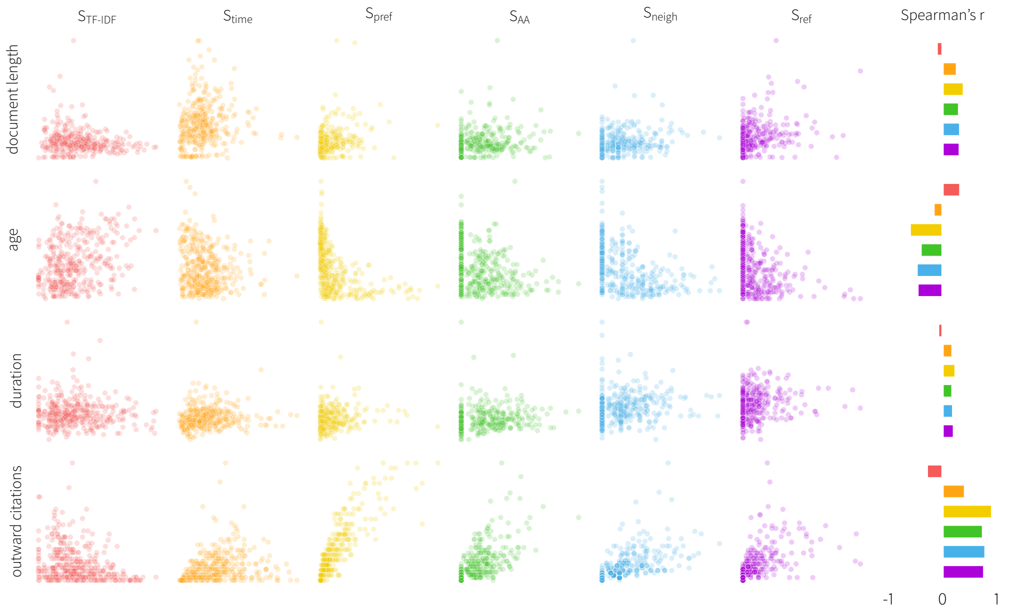


Figure S5: **Correlation between features and basic document properties**. Scatter plots show the correlation between features and meta information of the cases. For the sake of visibility, each plot is an under sampling of the whole court, corresponding to 5% of randomly chosen cases. The related Spearman's $r$ is displayed in the last column.

on its outgoing links, on which the aggregation is performed. Therefore, the more links a case has, the higher the chances are to observe extreme feature values, which result in a positive correlation of references and features.

## 2.2 Restricted training

Throughout the paper we use the classifier to estimate the significance of features through their predictive power and importance. Temporal trends are obtained by aggregating the network up to a year $Y_1$ and then training (and evaluating) the classifier on the links appearing between years $Y_1$ and $Y_2$. That is, classification results for year 1997 are based on training of the model on the outward citations of cases between 1992 and 1997 with all previous cases and citations already present in the network. The predictive power of the features is estimated by calculating the area under the receiver-operator

curve (ROC-AUC) using the raw feature values as well as training the model with a single feature.

## 2.3 Performance

Here we discuss the various aspects of the performance of our model that goes beyond the rank distribution shown in the main manuscript. Before evaluating the classifier, we carried out a grid search for the following hyper parameters (numbers in parentheses denote the optimal values): maximum number of features (4); maximum depth of trees (10) and minimum samples for a leaf (10). If not stated otherwise, we use a random forest of 1 000 trees for each analysis. The overall performance of the model is shown in Fig. S6, where we plot the temporal change of different metrics: accuracy, area under the receiver-operator curve (ROC-AUC), and $F_1$ score. In each case, we indicate the performance of a random classifier taking the imbalance present in the data into consideration. For $F_1$, each data point corresponds to the highest observed value of $F_1$ on the precision-recall curve. All metrics indicate that the model far exceeds the random classifier, and performance is robust over the years, and is not affected by the relatively large imbalance (1:1000). ROC-AUC is above 0.95 throughout the period of interest, and $\mathrm{F}_1$ also outperforms random classification by orders of magnitude. Performance can be measured at the level of cases as well, and a natural question is whether the efficiency of link prediction depends on the individual case. However, the metrics above are not suitable for single case predictions, as we only have a few number of observations, that is, the outgoing links of a case. Therefore, we calculate the relative rank of an existing link, when compared to all non-existing ones. More precisely, for each link, we find its rank in the list of all sorted non-links. If a prediction is efficient, the real link is positioned among the top ones, resulting in a low rank, and vice versa. Then, for each case the performance is simply the median of the rank over its outgoing links. Figure. S7 displays the correlations between inverse median rank and details of the case. Note that since low rank represents high performance, we plot the inverse of the ranks to have positive correlations whenever a case property correlates with performance. The most pronounced correlation is observed with age of the case: since early cases have fewer outgoing links, this sets a natural upper bound on the rank that can be achieved. The rest of the document details do not exhibit significant correlation, however, it is worth to mention that surprisingly, outward citations is negatively correlated with

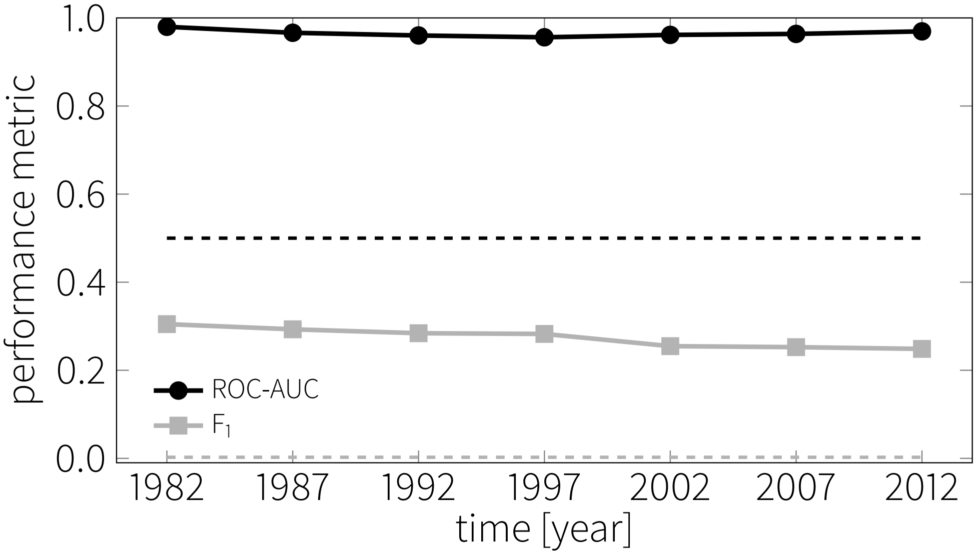


Figure S6: **Overall performance of the model**. Curves represent the performance of random forest classifiers trained on links present in the time periods corresponding to the previous four years of a specific year.

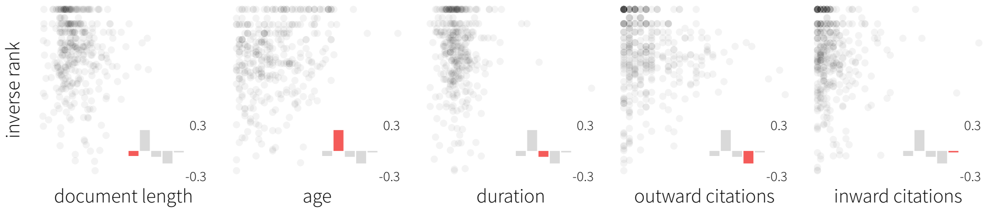


Figure S7: **Performance over cases**. Scatter plots of the inverse case ranks versus various case details. Histograms in the inset show the Spearman correlation, with the corresponding case property being highlighted. For sake of visibility, inverse rank is shown on a logarithmic scale.

performance.

## 2.4 Feature behavior

Here we investigate the behavior of the features with respect to various metrics:

> **Feature importance**: importance is defined by the Gini importance in the random forest classifier [1]. It measures the average decrease in the Gini impurity, each time a specific feature is used in the decision tree. In other words, importance is high if the feature contributes to many decisions that differentiate the output of the tree at a high level.
>
> **Random forest ROC-AUC**: area under the receiver-operator curve after training a random forest classifier using a single feature. This metric is insensitive of imbalance.
>
> **Random forest $\mathbf{F}_1$**: double harmonic mean of the precision and recall of a random forest classifier using a single feature. This metric is sensitive of the imbalance in the data. When reporting results, we always use the threshold which provides the best value for $F_1$.

Summary of the trends for the above four descriptors are shown in Fig. S8. Feature importance decreases over time for all features pertaining to content, the most drastic decrease is observed in $S_{\mathrm{TF-IDF}}$ and $S_{\mathrm{pref}}$. On the other hand, structural features show an increasing or steady importance with

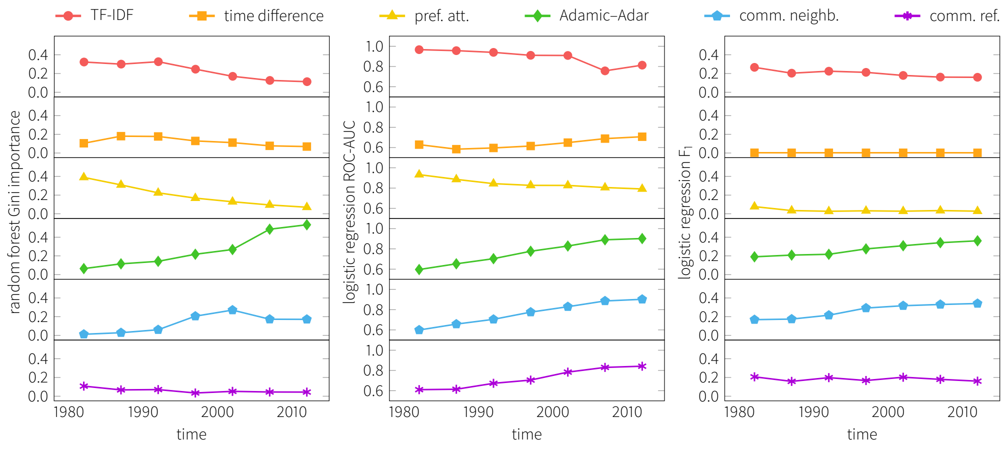


Figure S8: **Importance and performance trends of the six features**. Reported are the relative Gini importance (normalized by the initial value) of the features during random forest classification, and logistic regression ROC-AUC and $F_1$. Importance values are obtained from a random forest classifier using 1 000 trees.

$S_{\text{AA}}$ and $S_{\text{neigh}}$ increasing by 400% and 200% respectively. With respect to individual predicting power, performance of the logistic regression displays more profound differences: with the exception of ROC-AUC for $S_{\text{time}}$, all contextual features show vanishing efficiency compared to the improvement of structural features (ROC-AUC); or they (contextual properties) score below the structural ones ($F_1$). Note that in case of $F_1$, the random classifier has a performance of 0.0025 considering the imbalance present in the data.

# 3 Robustness

The problem of predicting links in a growing network is complex and multiple aspects of the actual system at hand affects performance and the behavior of the classifier. A variety of different mechanisms are in effect, each of them affecting the results differently: the growth of the network poses a non-trivial restriction on the structure; the inherent class imbalance due to the different number of potential and actual links; the changing size of the training set due to the increasing number of cases. Measuring or estimating

the combined effect of these aspects is not straightforward and is not within the scope of the current study. However, to gain a basic understanding of how our observations are influenced by the above characteristics, here we address the robustness of our results.

## 3.1 Class imbalance

When predicting existing references among cases, the classifier is trained and evaluated using all potential links. That is, for a case in year $y$ from the set of all cases $\mathcal{C}_{\mathrm{y_1,y_2}}$ between years $y_1$ and $y_2$, we consider all possible references to cases in years $y' < y$. The true positives and true negatives therefore constitute all possible time-respecting references from $\mathcal{C}_{\mathrm{y,y'}}$ to previous cases. Naturally, the number of potential links is magnitudes of order higher than the number of actual links, resulting in a large class imbalance of 1:1000. Interestingly, the imbalance is constant over the period between 1970-2012, meaning that the number of cases per citation grows with time. As shown in section Performance, the performance of the classifier is far better than the random classifier, and it shows a steady trend over the years irrespective of the metrics used to evaluate it. In order to address the problem of imbalance, and to ensure that the observed effects are not merely the outcome of some features favoring imbalance we report both ROC-AUC and $\mathrm{F}_1$ for the full classifier as well as for the single feature logistic regression. In all cases, the trends are similar with the structural features significantly outperforming contextual features.

Furthermore, in Fig. S9 we plot the raw ROC along with the precision-recall curves, as the latter is known to be a more informative evaluator for binary classification in imbalanced data sets [2]. The surprisingly high performance of the prediction is clear and both curves indicate that the observed predictability of the court is not an artifact of the imbalance but it is indeed related to structure within both content and structure.

## 3.2 Network growth

Some of the features, especially the structural ones are strongly influenced by the growth of the citation network. Each time a new case enters the network, it refers to already existing cases, either from previous years or from the same year. Therefore, a natural question arises whether the observed trends with respect to the various features are due to these restrictions of evolution

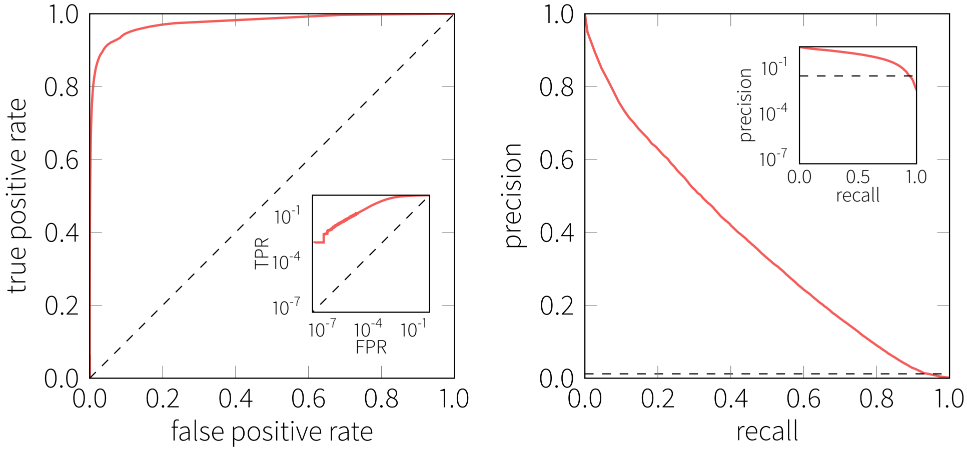


Figure S9: **Performance curves of the link prediction**. Plots show the ROC (left) and precision-recall curves (right) of the link prediction. Red curves correspond to our model, dashed black line is the baseline marking the random classifier. Insets show the same curves on a log-log (ROC) and log-lin (precision-recall) scales.

or inherent of the court network. To answer this question, we compare the temporal results to an uncorrelated network that is obtained by modifying the original citation graph via rewiring its directed links in a degree-conserving and time-respecting manner. A single step in the rewiring process chooses two links randomly and swaps their end points if the resulting links still point backwards in time, or have end nodes in the same year. On average we carried out at least 5 swaps per link. Feature related trends are summarized in Fig. S10, where we report the feature importance, ROC-AUC and $F_1$ metrics of single feature logistic regressions. All feature importance values in the uncorrelated network show significantly deviating trend relative to the true network. Although it seems that contextual features gain importance over the years, but the absolute value of importance values is still insignificant (and the improvement is merely noise) as the overall performance of the classifier in the uncorrelated network is the same as the random classifier in the original network (ROC-AUC of 0.5 and $\mathrm{F}_1$ of 0.0025). ROC-AUC trends for logistic regressions show striking differences with the only significant performance observed by $S_{\mathrm{pref}}$: naturally, this feature remains the only one being able to grasp any structure in the network. However, in general, all features show vanishing performance. These results suggest that the observed trends are not the outcome of the simple growing dynamics but characterize a particular behavior of the court over time.

## 3.3 Training set

As the court grows in number of cases as well as citations, the input data to our classifier also becomes larger. In other words, there is more data available on which to train the model, which in theory may affect the prediction's overall performance, especially the results for single features. The major result of our analysis, that is, the qualitative difference in the behavior of performance with respect to different features, relies on the fact that some properties of the court increase in importance over time with respect to inferring references accurately while others remain constant. Thus, it is imperative that we address the question whether the trends are due to the increasing data quality, with special regard to structural features. To address this concern, we repeated the prediction with multiple under-sampled data sets: in each 5 year period, we sampled 432 970 of links, which corresponds to the size of the data in the first period (1978-1982), results are summarized in Fig. S11. We find that feature importance values remain the same in the under sam-

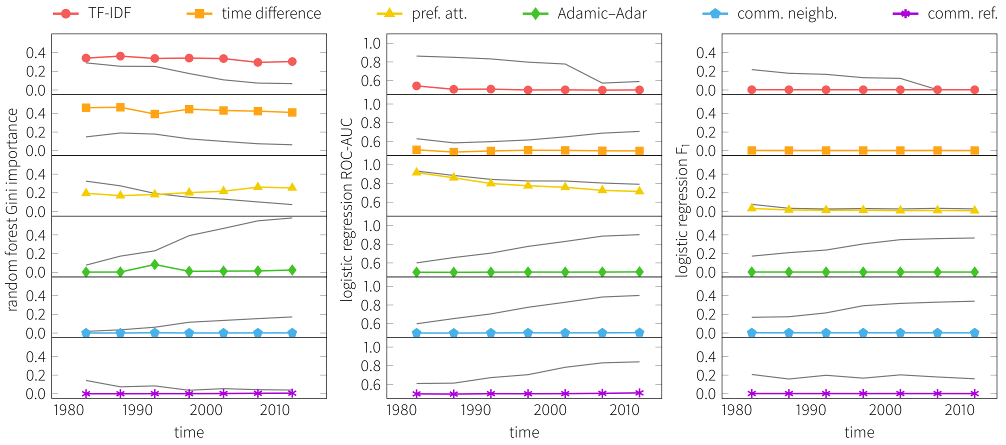


Figure S10: **Feature trends observed in the uncorrelated model**. Reported are the random forest feature importance, logistic regression ROC-AUC and logistic regression $F_1$. Color lines show the results in the uncorrelated model, gray lines indicate the trends in the original network.

pled data set, only small deviations are observed in the last years. Also, performance of the contextual features are robust against under-sampling, only $S_{\mathrm{TF-IDF}}$ displays a slightly better ROC-AUC. In overall, we conclude that our findings are not affected by the increasing size of training set.

# 4 Interpreting predictions

One of the most straightforward application of our results is a recommender system that aims to support legal practitioners. Given a case in the CJEU, such a system would provide potential references that are corroborated by the content of the cases and take the underlying structure of the court into consideration. To evaluate the validity of predictions based on automatization, here we provide qualitative analysis and discussion of the output of our model. We start by the best predictions (true positives) and later we consider the most extreme cases when the classifier failed: predictions that score high in our model but are nonetheless absent in the court (false positives) along with existing references that received the lowest probability (false negatives). The results are shown in Tables S1-S3.

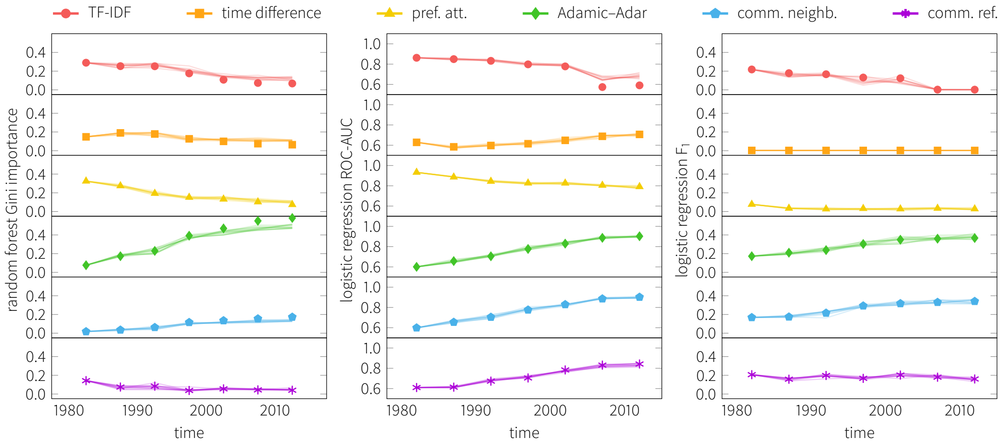


Figure S11: **Feature trends in the under sampled data set.** Reported are the random forest feature importance, logistic regression ROC-AUC and logistic regression $F_1$. Transparent color lines show the results in the under sampled data set, dots correspond to the trends in the original network.

| Citing case | Cited case | Description |
|---|---|---|
| Case 811/79 - Amministrazione delle finanze dello Stato v Ariete SpA. | Case 61/79 - Amministrazione delle finanze dello Stato v Denkavit italiana Srl. | The citing case is concerned with a Preliminary Question regarding the repayment of sums levied as custom duties – similar to the cited case. |
| Case C-343/96 - Dilexport Srl v Amministrazione delle Finanze dello Stato. | Case C-228/96 - Aprile Srl, in liquidation, v Amministrazione delle Finanze dello Stato. | The cited case is of nearly identical subject matter to the citing case – both concerning the time-limits on repayment of custom duties. |
| Case C-397/11 - Erika Jőrös v Aegon Magyarország Hitel Zrt. | Case C-488/11 - Dirk Frederik Asbeek Brusse and Katarina de Man Garabito v Jahani BV. | Both cases concern Directive 93/13/EEC on unfair terms in consumer contracts. The cited case applies the principle of equivalence, as the citing case should as well. |
| Case C-443/12 - Actavis Group PTC EHF and Actavis UK Ltd v Sanofi. | Case C-484/12 - Georgetown University v Octrooicentrum Nederland. | The citing case is questioning the correct application of the principles set down in the cited case – concerning protection certificates for medicinal products and the conditions for obtaining them. |
| Case C-305/09 - European Commission v Italian Republic. | Case C-304/09 - European Commission v Italian Republic. | The prescribed time limit set for implementing the measures in the cited case was not fulfilled in the citing case – concerning an unlawful State Aid Scheme. |

Table 1: Table showing some example of predicted references with the highest probability, indicating a high confidence of the model.

| **Citing case** | **Cited case** | **Description** |
|---|---|---|
| Case C-284/09 - European Commission v Federal Republic of Germany. | C-436/08 and C-437/08 - Haribo Lakritzen Hans Riegel Betriebs-gmbH (C-436/08) and Österreichische Salinen AG (C-437/08) v Finanzamt Linz. | While both cases deal with The Free Movement of Capital and Dividends Taxation, the citing case is concerned specifically with Directive 90/435, while the cited case is a preliminary reference regarding TFEU art. 63. |
| Case C-587/10 - Vogtländische Straßen-, Tief- und Rohrleitungsbau GmbH Rodewisch (VSTR) v Finanzamt Plauen. | Case C-409/04 - The Queen, on the application of Teleos plc and Others v Commissioners of Customs & Excise. | Both cases deal with Directive 77/388, article 28c(a), concerning the harmonisation of the laws of the Member States relating to turnover taxes. |
| Case C-379/05 - Amurta SGPS v Inspecteur van de Belastingdienst/Amsterdam. | Case C-374/04 - Test Claimants in Class IV of the ACT Group Litigation v Commissioners of Inland Revenue. | Both cases deal with article 56 EC, concerning treating dividends paid to companies established in another Member State less favourably than dividends paid to companies established in the Member State. |
| Case C-329/97 - Sezgin Ergat v Stadt Ulm. | Case C-237/91 - Kazim Kus v Landeshauptstadt Wiesbaden. | Both cases deal with Decision No 1/80 of the EEC-Turkey Council of Association, regarding the right of residence for a Turkish worker. |
| Case C-304/06 P - Eurohypo AG v Office for Harmonisation in the Internal Market (Trade Marks and Designs) (OHIM). | Case C-412/05 P - Alcon Inc. v European Union Intellectual Property Office. | Both cases deals with Community Trade Mark Law, including the distinctive characteristics of a mark. However, the case *that should have been cited* is concerned primarily with the admissibility of the appeal. |

Table 2: Table showing some example of references that were predicted by the model but are not present in the court.

| **Citing case** | **Cited case** | **Description** |
|---|---|---|
| Case 124/83 - Direktoratet for Markedsordningerne v SA Nicolas Corman et fils. | Joined cases 94-63 and 96-63 - Pierre Bernusset v Commission of the European Economic Community. | *Bernusset* is cited by the defendant as evidence of a general principle, that an authority which adopts measures affecting the persons concerned or which withdraws a favourable decision must bear the burden of proof itself. |
| Case C-181/95 - Biogen Inc. v Smithkline Beecham Biologicals SA. | Case 201/84 - Procureur de la République v Jean-Pierre Gontier. | The citation appears to be a clerical error in the case details – the citation in the actual judgment is Case C-201/84. |
| Case C-296/95 - The Queen v Commissioners of Customs and Excise, ex parte EMU Tabac SARL, The Man in Black Ltd, John Cunningham. | Case 170/83 - Hydrotherm Gerätebau GmbH v Compact del Dott. Ing. Mario Andreoli & C. Sas. | *Hydrotherm* is cited as an example of seeing subsidiaries of the same company and as such they can be regarded as forming part of the same economic entity, despite the fact that they are separate legal persons. |
| Case C-191/98 P - Georges Tzoanos v Commission of the European Communities. | Case 107/82 - Allgemeine Elektrizitäts-Gesellschaft AEG-Telefunken AG v Commission of the European Communities. | *AEG* is cited as an example of a case where the exclusion of certain commission documents is allowed. |
| Case C-95/12 - European Commission v Federal Republic of Germany. | Case 135/77 - Robert Bosch GmbH v Hauptzollamt Hildesheim. | *Bosch* is used an example how to interpret the particular wording of a decision – in this case the phrasing "In conjunction with" and "As well as". |

Table 3: Table showing some examples of references that are present in the court but were not found by the classifier.

# 5 Context and structure

The non-trivial interplay between the difficulty of prediction and the segregation of the court into sub-fields raises the question of whether the trends seen in $S_{\mathrm{TF-IDF}}$ do indeed reflect an essential aspect of the court. A steady $S_{\mathrm{TF-IDF}}$ performance suggests that although an exponentially growing number of cases would imply an increase in the difficulty of the prediction of links (due to the increasing noise/signal ratio), something which does not affect the efficiency of TF-IDF. On explanation for this behavior is that during the evolution of the court, legal principles are created and go out of use, creating a complex structure of topics. Specifically, the cases may aggregate in specific sub-fields, creating specialized groups of cases that are contextually similar. As a consequence, for each single case, there is a constant number of other cases dealing with the same legal issues. This interpretation is supported by Fig. S12, where we count the average number of cases within a $\Delta$ cosine distance of TF-IDF vectors (that is, the cosine similarity of the TF-IDF vectors is above $1 - \Delta$). For each year, we consider all new cases and calculate the average number of typically close cases, curves represent the trends at different distance thresholds. Indeed, it appears that the number of cases in close proximity of any case in TF-IDF space, is constant over time. On the contrary, if we allow documents to be far from any case in terms of topic, we see an increasing number of cases. Note that the fraction of case pairs closer than $\Delta = 0.6$ and $\Delta = 0.8$ is only 0.14% and 1.23%, meaning that the top curves are not merely the result of counting all cases in a growing network. The inset of Fig. S12 shows the distribution of TF-IDF restricted to cases in the denoted decades. As we can see, cases in the court tend to be further away from each other, however, the distribution saturates and the mode of the TF-IDF values becomes steady – marking the observation that each single case perceives a closer inner circle of similar cases and a larger distance shell of the rest of the network.

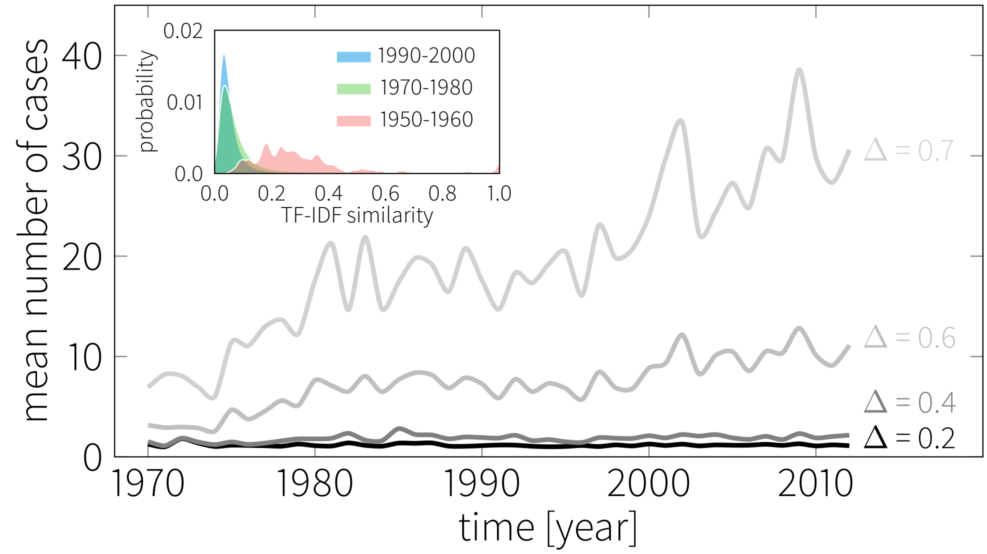


Figure S12: **Average number of topically close cases a single case perceives**. Curves represent the average number of cases with a TF-IDF similarity higher than $1 - \Delta$, as a function of time. Inset shows the distribution of TF-IDF values for all pairs of cases appearing in the respective decades.

# 6 Communities

We now explore another level of complexity within the court's network of citations. Specifically, we look at *communities* – groups of decisions with a high probability of internal citations and lower probability of linking to decisions outside the group [3, 4]. We stress that the communities are purely defined by the network structure, capturing groups of decisions that are densely interconnected, while weakly connected to the remainder of the network. There are many ways to find communities in complex networks [5]. Here, we use an information theoretical approach (the InfoMap algorithm [3]), which is considered state-of-the-art within network science, and has already been applied to characterize the community structure of the European Court [4]. Here, we analyze the content and citation patterns in the ten largest communities in detail; these communities account for 15% of all cases in the giant component.

## 6.1 Consistency

In order to validate that the communities are meaningful beyond capturing structural properties of the network, we first calculate the similarity of the written content of the cases within and outside communities, using the TD-IDF vectors describing each document (for more details, see Materials and Methods). Figure S13 shows the relative similarity between the communities (the size of each community is shown in the top panel). The blue tiles along the diagonal illustrate that cases within communities tend to have high similarity among their important words. Each tile in Fig. S13 represents the median similarity of cases between two communities, with the average over the entire matrix (over all pairs of communities) subtracted from each tile. Typical distribution of similarity values are shown on the right side of Fig. S13 where we plotted three examples for low (red), moderate (yellow) and high (green) similarity. If two communities have low average topical similarity, judgments within those communities have low similarity in the TF-IDF space as well, indicating that the important words in those documents tend to be different. We can therefore assume that two low-similarity communities revolve around different legal concepts. As a counterpoint, a high similarity represents high content-overlap indicating that judgments belong to similar legal fields. In Fig. S13, for each community, the similarity within the community is found to be the highest among all similarities, in-

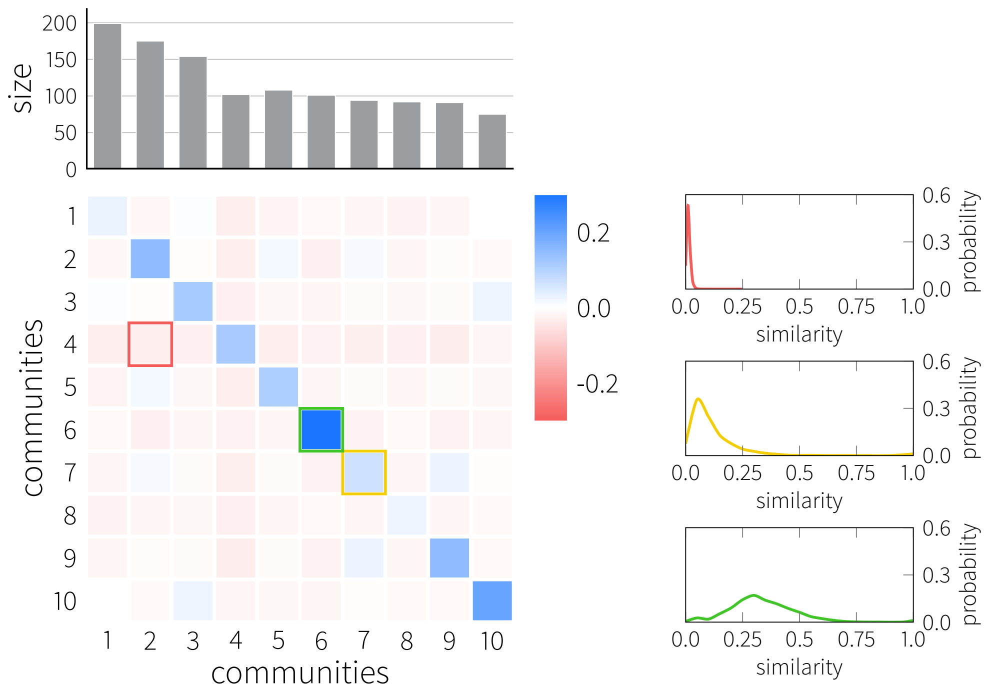


Figure S13: **Nodal feature similarity within and between communities**. Heat map similarity matrix shows the median cosine similarity of TF-IDF vectors between cases of indicated communities, normalized by the average value of the tiles. The size of each communities is shown at the top of the heat map. Figures on the left display three typical distribution of similarity measures highlighted in the heat map.

dicating a high level of coherence of cases inside the communities.

We also explore two other methods to evaluate the validity of the communities: referential density between communities and the overlap in the content of the member cases. Figure. S14 displays the comparison of the communities. In Fig. S14a shows the number of references within and between communities: the heat map is dominated by the diagonal indicating the majority of the citations remain inside the communities, suggesting that communities found are robust and denote well-defined separated parts of the court from a network perspective. With respect to content, we compare the related articles of the Treaty: for each case we determine the frequency of

Treaty articles mentioned in the corresponding document, and the average cosine similarity of these frequencies between all cases of two communities is calculated (see Fig. S14b). We note that the distribution is more homogeneous than before, however, the average similarity of cases within communities are among the top two in all communities, that is, the diagonal includes the highest or the second highest similarity in each row/column.

## 6.2 Legal content of each community

In determining the legal content of each community, we selected the 10 most cited cases within the community and studied those to determine to which extent they were concerned with same (or similar) kind of legal problem or whether they were concerned with a variety of legal content. We found that legal problems in the communities did indeed concern related legal issues; typically each community focused on a Treaty on the Functioning of the European Union (TFEU).

**Community 1 (199 cases): Competition Law.** The top cases in this community focus on legal issues related to EU competition law. (See SI for full details on communities and related treaty articles)

**Community 2 (175 cases): Free Movement.** This community focuses on legal issues related to the EU's rule on free movement of persons, services and capital and the associated right of establishment, that allows undertakings to freely conduct business across the EU.

**Community 3 (154 cases): State Aid and Markets.** In this community, the top cases are concerned with issues related to the EU's prohibition on state aid that distorts market competition.

**Community 4 (102 cases): Custom Tariffs/Preliminary rulings procedure.** The focus of this community are cases that dealt are with by the CJEU under the preliminary rulings procedure.

**Community 5 (108 cases): Indirect taxation/regulatory form** This community deals with the form of regulatory instruments that may be used by the EU institutions (regulations, directives, decisions, recommendations and opinions).

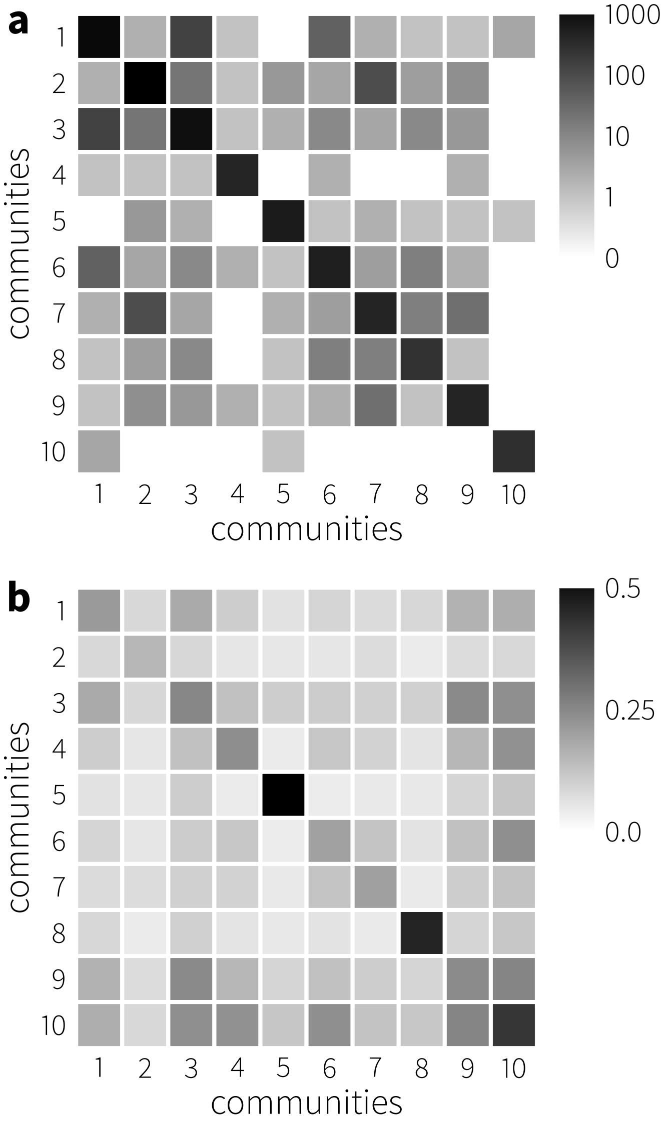


Figure S14: **Connectivity and similarity between communities**. Number of links between communities (a) and the average overlap of article-frequency vectors of the member cases (b).

**Community 6 (101 cases): Jurisdiction/General Court.** The top cases in this community relate to jurisdiction of the EU's General Court to hear and determine at first instance actions or proceedings of a specific kind.

**Community 7 (94 cases): Discrimination Based on Nationality.** This cluster of cases, judging from the content of the top cases, deals predominantly with issues in regards to the prohibition in EU law against discrimination on grounds of nationality and the rights of EU citizens to freely move and reside within the territory of the member states in the EU.

**Community 8 (92 cases): Restrictions on Import.** This cluster of cases mostly deal with the prohibition in EU law against quantitative restrictions on imports (raised by one EU member state against another) and against all measures that have an equivalent effect.

**Community 9 (91 cases): Gender Discrimination** These cases deal with issues in regards to the prohibition against gender discrimination in regards to pay for work.

**Community 10 (75 cases): Distribution of Powers** The top cases in this community revolve around the distribution of powers between the European Union and the members states.

## 6.3 Activity over time within communities

We observe that all communities show increased activity after 1990 and most communities reflect the general growth-patterns observed in the full network. There are, however, individual differences. Here we discuss in detail difference between two communities: *Competition Law* and *Restrictions on Import*, as an illustrative example. The former community has a strong recent peak indicating a steep rise in adjudicative activity in this field. The TFEU art. 101 sets out a prohibition on agreements between undertakings, on decisions by associations of undertakings and on concerted practices which may affect trade between Member States and which have as their object or effect the prevention, restriction or distortion of competition within the internal market. A likely explanation for the rise of activity in the Competition Law community could be related to an increased monitoring of the markets by the

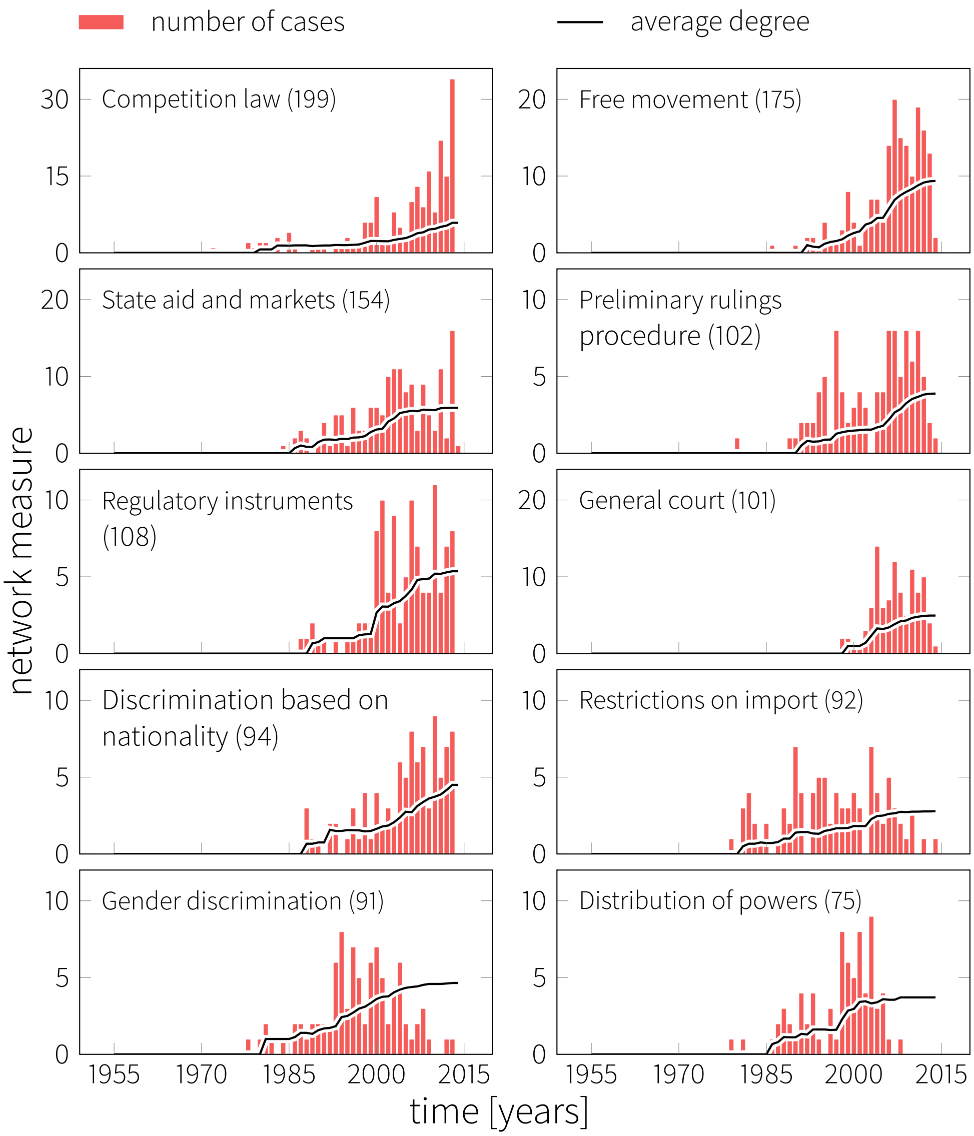


Figure S15: **Evolution of communities**. For each of the ten communities, we report the number of cases (red bars) as well as the average degree (black line). The numbers next to the communities' name denote the number of cases in the group.

European Commission, which could have led to more findings of concerted practices or the like, which in turn could produce more cases brought before the court.

The community *Restrictions on Import* has its peak of intensity between 1990 and 2000. This community revolves around the prohibition in EU law against quantitative restrictions on imports and against all measures that have an equivalent effect (TFEU art. 34). A likely explanation for the activity curve in this community is that the Court has managed to develop clear standards and has consistently applied these standards over many different cases for a extended period of time, which in turn has led to an internalization of these standards in member state behavior, reducing the need for resolving disputes between member states.

As illustrated through these concrete examples, our findings can be used as a starting point for inquiring into the specific development of legal areas within EU law. The likely explanations for the developments in *Competition Law* and *Restrictions on Import* we set out above can be seen as working hypotheses, which could set the foundation for further exploration.

## 6.4 Community fingerprints

Connecting the structural analysis to the recommender system by studying the behavior of the nodal and structural features within each community, we find non-trivial interaction patterns.

To make this connection, we first train the classifier in the aggregated network consisting of verdicts in the top ten communities combined. This allows us to obtain an appropriate baseline of feature importance. Once the baseline has been determined, we train the classifier on each community separately, a process which results in the computation of feature-importance based on links solely within each single community. The error of the feature importance in each community is calculated by $\sqrt{(\partial_I f)^2\sigma_I^2 + (\partial_B f)^2\sigma_B^2}$ with $f = \frac{I-B}{B}$, where $I$ is the importance obtained by training the model in a community, and $B$ is the baseline importance, that is, training the model in the union of all ten communities. The results of these community-specific feature importance values are shown in Fig. S16, where we plot the deviation of community-wise importance values relative to the baseline feature importance for all ten communities.

We find that the importance patterns are somewhat different across the

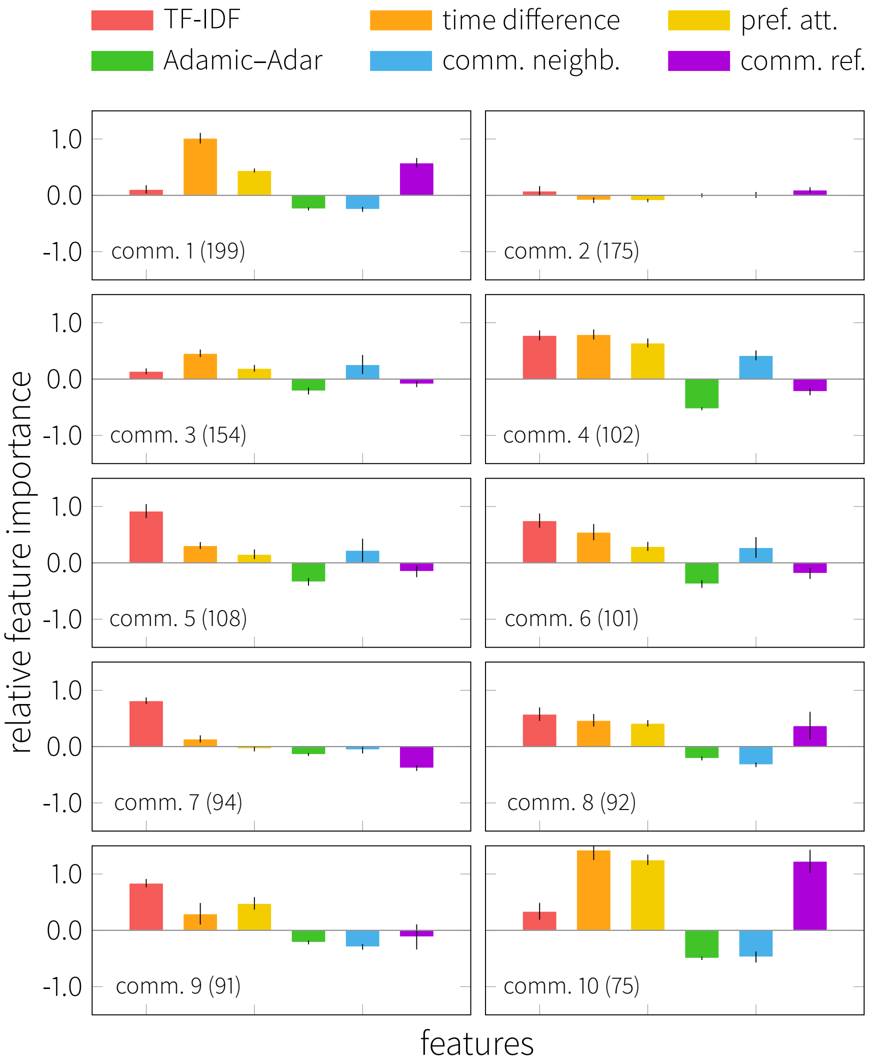


Figure S16: **Community fingerprints**. For each community, histograms show the difference in feature importance between the community trained classifier and the globally trained one. Black strokes mark the error of the importance values defined by the standard deviation over the cases in the community.

ten communities, with each community being characterized by a particular pattern of feature importance. We call these observed deviations that are specific to each community the 'predictive fingerprint' of each community. Structural features are over-represented in the communities of *Free Movement*, *Jurisdiction/General Court*, and *Gender discrimination* with an under-representation of nodal features. On the contrary, nodal properties are more relevant in *Competition Law*, *Custom Tariffs/Preliminary rulings procedure*, and *Distribution of Powers*. Other communities exhibit deviations only to a lower extent. Furthermore, note that the groups *Indirect taxation/regulatory form*, *Discrimination Based on Nationality* and *Restrictions on Import* share high level of similarity in their predictive fingerprints, described as moderate positive TF-IDF importance, lower than average time difference and preferential attachment importance, and a high common neighbors or common referrers importance.

## 6.5 Algorithm performance within communities

If we measure the performance of a classifier train on each community individually. More precisely, we estimated the efficacy of the classifier in two ways. First, we calculated the median rank of real citations among non-existent citations for each case, that is, how well the existing reference is positioned when compared to the all potential links. Note that in this comparison we did not include other existing links, so that the median rank is not biased towards low values for hubs (cases with saliently large citations). Second, for each community we calculated the fraction of links that were ranked among the top 20 links predicted by the classifier. Therefore high performance is associated with a low median rank and a high median fraction of top 20 links, that is, real citations are among the ones that score high in the model. Results are shown in Fig. S17, with the median rank and fraction of links among the top 20 predictions relative to the baseline. As baseline we considered the same performance measures but with a classifier trained in the aggregate network of all ten communities. This way we effectively assess how much information we gain in total when communities are treated as isolated. To understand how this influences the algorithm consider two competing mechanisms. On one hand a single community contains less information as we only consider citations inside the community, we expect that all things equal, this will result in a decrease in the performance of the classifier. On the other hand, the fact that cases inside a community are expected to be more similar

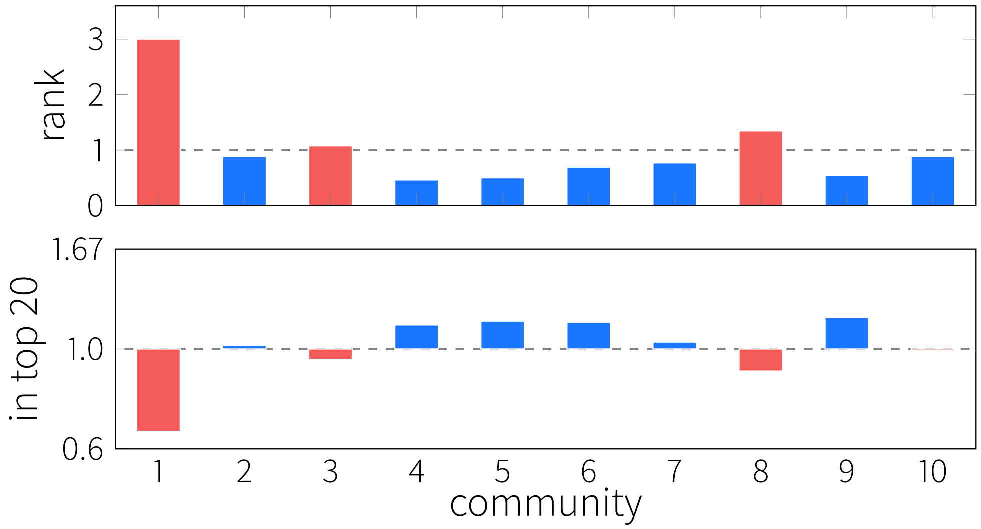


Figure S17: **Predictability of communities**. Bar charts show the median rank (top) and median fraction of existing citations among the top 20 when each being compared to all other links (in log scale for better comparison). All values are relative to the baseline (dashed line), blue color marks communities that are more predictable, red color denotes lower prediction performance.

to each other and tend to follow the same citation tradition, may result in an improvement in their predictability. As a general observation, we see that both median rank and top 20 fraction display similar trends: communities 1, 3 and 8 are less predictable on their own, while prediction performs rather well for the rest of the communities.

## 6.6 Feature relevance

In the main text we explored the way the random forest model uses the different features, that is, what features are used in the different levels of the individual decision trees. We showed that in the early years of the court, nodal features such as content or age of the cases influence the decisions at every level of the trees, marking the fact that using these features results in the most optimal split of the data. As the court evolved, however, structural features began to dominate the random forest, especially the top of the trees

where the highest influence is exerted. The same analysis can be performed with respect to the models trained at the community level, results are shown in Fig. S18. The results suggest that the decision trees use the features in the communities in a way that is similar to the global network: the top of the trees are dominated by structural related features and as the decision making process propagates through the trees, more and more nodal features are used. In other words, the court network exhibits a fractal-like behavior where structural properties of any subgraph are used to rough estimate of the existence of a link whereas nodal features are considered during fine-tuning once the overall structure is detected.

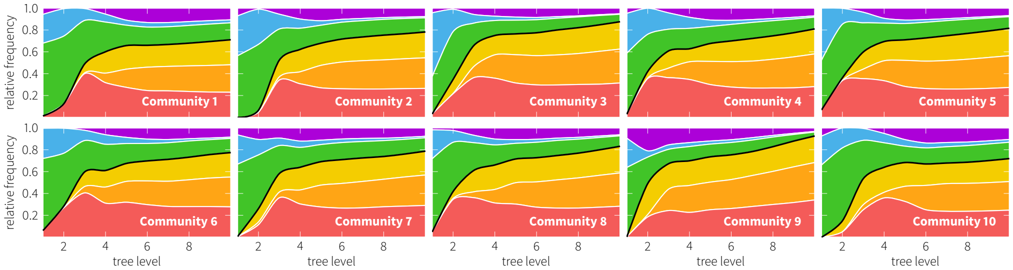


Figure S18: **Details of feature usage inside the decision trees when trained in each community**. Curves show the fraction of decision nodes in the decision trees that use a specific feature in different levels of the trees (they add up to one). For each feature, we calculate the number of (internal) decision nodes that make the split based on the value of that feature, normalized by the total number of nodes in that level. Results are averaged over all trees in a random forest and over 5 independent forests. Black lines indicate the boundary between nodal (lower) and structural (upper) features. Reach figure corresponds to a model that is trained on cases of a single community in 2008.

# 7 Table of treaty provisions

## 7.1 Community 1: Competition Law

The top 10 cases in this community focus on legal issues related to EU competition law. TFEU art. 101 sets out a prohibition on agreements between

undertakings, on decisions by associations of undertakings and on concerted practices which may affect trade between Member States and which have as their object or effect the prevention, restriction or distortion of competition within the internal market.

**TFEU art. 101 (ex TEC art. 81)**

1. The following shall be prohibited as incompatible with the internal market: all agreements between undertakings, decisions by associations of undertakings and concerted practices which may affect trade between Member States and which have as their object or effect the prevention, restriction or distortion of competition within the internal market, and in particular those which: (a) directly or indirectly fix purchase or selling prices or any other trading conditions; (b) limit or control production, markets, technical development, or investment; (c) share markets or sources of supply; (d) apply dissimilar conditions to equivalent transactions with other trading parties, thereby placing them at a competitive disadvantage; (e) make the conclusion of contracts subject to acceptance by the other parties of supplementary obligations which, by their nature or according to commercial usage, have no connection with the subject of such contracts.

2. Any agreements or decisions prohibited pursuant to this Article shall be automatically void.

3. The provisions of paragraph 1 may, however, be declared inapplicable in the case of: - any agreement or category of agreements between undertakings, - any decision or category of decisions by associations of undertakings, - any concerted practice or category of concerted practices, which contributes to improving the production or distribution of goods or to promoting technical or economic progress, while allowing consumers a fair share of the resulting benefit, and which does not: (a) impose on the undertakings concerned restrictions which are not indispensable to the attainment of these objectives; (b) afford such undertakings the possibility of eliminating competition in respect of a substantial part of the products in question.

## 7.2 Community 2: Free Movement

The top 10 cases in this community focus on legal issues related to the EU's rule on free movement of persons, services and capital and the associated right of establishment, that allows undertakings to freely conduct business across the EU. The most relevant treaty provisions in this regard is TFEU 63 and 49.

### TFEU art. 49 (ex TEC art 43)

Within the framework of the provisions set out below, restrictions on the freedom of establishment of nationals of a Member State in the territory of another Member State shall be prohibited. Such prohibition shall also apply to restrictions on the setting-up of agencies, branches or subsidiaries by nationals of any Member State established in the territory of any Member State. Freedom of establishment shall include the right to take up and pursue activities as self-employed persons and to set up and manage undertakings, in particular companies or firms within the meaning of the second paragraph of Article 54, under the conditions laid down for its own nationals by the law of the country where such establishment is effected, subject to the provisions of the Chapter relating to capital.

### TFEU art. 63 (ex TEC art. 56)

1. Within the framework of the provisions set out in this Chapter, all restrictions on the movement of capital between Member States and between Member States and third countries shall be prohibited.

2. Within the framework of the provisions set out in this Chapter, all restrictions on payments between Member States and between Member States and third countries shall be prohibited.

## 7.3 Community 3: State Aid and Markets

The top 10 cases in this community are concerned with issues related to the EU's prohibition on state aid that distorts market competition. The rules relating to this prohibition and the procedures for surveying respect for free competition are set out in TFEU art. 107 and 108.

**TFEU art. 107 (ex TEC art. 87)**

1. Save as otherwise provided in the Treaties, any aid granted by a Member State or through State resources in any form whatsoever which distorts or threatens to distort competition by favouring certain undertakings or the production of certain goods shall, in so far as it affects trade between Member States, be incompatible with the internal market.

2. The following shall be compatible with the internal market: (a) aid having a social character, granted to individual consumers, provided that such aid is granted without discrimination related to the origin of the products concerned; (b) aid to make good the damage caused by natural disasters or exceptional occurrences; (c) aid granted to the economy of certain areas of the Federal Republic of Germany affected by the division of Germany, in so far as such aid is required in order to compensate for the economic disadvantages caused by that division. Five years after the entry into force of the Treaty of Lisbon, the Council, acting on a proposal from the Commission, may adopt a decision repealing this point.

3. The following may be considered to be compatible with the internal market: (a) aid to promote the economic development of areas where the standard of living is abnormally low or where there is serious underemployment, and of the regions referred to in Article 349, in view of their structural, economic and social situation; (b) aid to promote the execution of an important project of common European interest or to remedy a serious disturbance in the economy of a Member State; (c) aid to facilitate the development of certain economic activities or of certain economic areas, where such aid does not adversely affect trading conditions to an extent contrary to the common interest; (d) aid to promote culture and heritage conservation where such aid does not affect trading conditions and competition in the Union to an extent that is contrary to the common interest; (e) such other categories of aid as may be specified by decision of the Council on a proposal from the Commission.

**TFEU art. 108 (ex TEC art. 88)**

1. The Commission shall, in cooperation with Member States, keep under constant review all systems of aid existing in those States. It shall propose to the latter any appropriate measures required by the progressive development or by the functioning of the internal market.

2. If, after giving notice to the parties concerned to submit their comments, the Commission finds that aid granted by a State or through State resources is not compatible with the internal market having regard to Article 107, or that such aid is being misused, it shall decide that the State concerned shall abolish or alter such aid within a period of time to be determined by the Commission. If the State concerned does not comply with this decision within the prescribed time, the Commission or any other interested State may, in derogation from the provisions of Articles 258 and 259, refer the matter to the Court of Justice of the European Union direct. On application by a Member State, the Council may, acting unanimously, decide that aid which that State is granting or intends to grant shall be considered to be compatible with the internal market, in derogation from the provisions of Article 107 or from the regulations provided for in Article 109, if such a decision is justified by exceptional circumstances. If, as regards the aid in question, the Commission has already initiated the procedure provided for in the first subparagraph of this paragraph, the fact that the State concerned has made its application to the Council shall have the effect of suspending that procedure until the Council has made its attitude known. If, however, the Council has not made its attitude known within three months of the said application being made, the Commission shall give its decision on the case.

3. The Commission shall be informed, in sufficient time to enable it to submit its comments, of any plans to grant or alter aid. If it considers that any such plan is not compatible with the internal market having regard to Article 107, it shall without delay initiate the procedure provided for in paragraph 2. The Member State concerned shall not put its proposed measures into effect until this procedure has resulted in a final decision.

4. The Commission may adopt regulations relating to the categories of

State aid that the Council has, pursuant to Article 109, determined may be exempted from the procedure provided for by paragraph 3 of this Article.

## 7.4 Community 4: Custom Tariffs/Preliminary rulings procedure

Top 10 cases in this community concern cases that are dealt with by the CJEU under the preliminary rulings procedure. This procedure is set out in TFEU art. 267. The substantive content of most of the cases in the community are concerned with cases relating to the prohibition against leveraging customs tariffs, which are regulated in TFEU art. 28 and 30.

#### TFEU art. 267 (ex TEC art. 234)

The Court of Justice of the European Union shall have jurisdiction to give preliminary rulings concerning: (a) the interpretation of the Treaties; (b) the validity and interpretation of acts of the institutions, bodies, offices or agencies of the Union; Where such a question is raised before any court or tribunal of a Member State, that court or tribunal may, if it considers that a decision on the question is necessary to enable it to give judgment, request the Court to give a ruling thereon. Where any such question is raised in a case pending before a court or tribunal of a Member State against whose decisions there is no judicial remedy under national law, that court or tribunal shall bring the matter before the Court. If such a question is raised in a case pending before a court or tribunal of a Member State with regard to a person in custody, the Court of Justice of the European Union shall act with the minimum of delay.

#### TFEU art. 28 (ex TEC Article 23)

1. The Union shall comprise a customs union which shall cover all trade in goods and which shall involve the prohibition between Member States of customs duties on imports and exports and of all charges having equivalent effect, and the adoption of a common customs tariff in their relations with third countries. 26.10.2012 EN Official Journal of the European Union C 326/59.

2. The provisions of Article 30 and of Chapter 3 of this Title shall apply to products originating in Member States and to products coming from third countries which are in free circulation in Member States.

**TFEU art. 30 (ex TEC art 25)**

Customs duties on imports and exports and charges having equivalent effect shall be prohibited between Member States. This prohibition shall also apply to customs duties of a fiscal nature.

## 7.5 Community 5: Indirect taxation/regulatory form

The top 10 cases in this community deal with the form of regulatory instruments that may be used by the EU institutions (regulations, directives, decisions, recommendations and opinions). The substantive content of these cases relates mostly to indirect taxation, but in this case under secondary regulation, i.e. Council Directive 2006/112/EC of 28 November 2006 on the common system of value added tax (adopted under TFEU article 113). It should be noted that there is an overlap between customs duties (Custom Tariffs/Preliminary rulings procedure community) and VAT/indirect tax.

**TFEU art. 288 (ex TEC art. 249)**

To exercise the Union's competences, the institutions shall adopt regulations, directives, decisions, recommendations and opinions. A regulation shall have general application. It shall be binding in its entirety and directly applicable in all Member States. A directive shall be binding, as to the result to be achieved, upon each Member State to which it is addressed, but shall leave to the national authorities the choice of form and methods. A decision shall be binding in its entirety. A decision which specifies those to whom it is addressed shall be binding only on them. Recommendations and opinions shall have no binding force.

**TFEU article 113 (ex Article 93 TEC)**

The Council shall, acting unanimously in accordance with a special legislative procedure and after consulting the European Parliament and the Economic and Social Committee, adopt provisions for the harmonisation of legislation concerning turnover taxes, excise duties and other forms of indirect taxation

to the extent that such harmonisation is necessary to ensure the establishment and the functioning of the internal market and to avoid distortion of competition.

## 7.6 Community 6: Jurisdiction/General Court

The top 10 cases in this community relate to jurisdiction of the EU's General Court to hear and determine at first instance actions or proceedings of a specific kind (TFEU art. 256). The substantive content of the cases revolve around EU trademark law.

**TFEU art. 256 (ex TEC art. 225)**

1. The General Court shall have jurisdiction to hear and determine at first instance actions or proceedings referred to in Articles 263, 265, 268, 270 and 272, with the exception of those assigned to a specialised court set up under Article 257 and those reserved in the Statute for the Court of Justice. The Statute may provide for the General Court to have jurisdiction for other classes of action or proceeding. Decisions given by the General Court under this paragraph may be subject to a right of appeal to the Court of Justice on points of law only, under the conditions and within the limits laid down by the Statute.

2. The General Court shall have jurisdiction to hear and determine actions or proceedings brought against decisions of the specialised courts. Decisions given by the General Court under this paragraph may exceptionally be subject to review by the Court of Justice, under the conditions and within the limits laid down by the Statute, where there is a serious risk of the unity or consistency of Union law being affected.

3. The General Court shall have jurisdiction to hear and determine questions referred for a preliminary ruling under Article 267, in specific areas laid down by the Statute. Where the General Court considers that the case requires a decision of principle likely to affect the unity or consistency of Union law, it may refer the case to the Court of Justice for a ruling. Decisions given by the General Court on questions referred for a preliminary ruling may exceptionally be subject to review by the Court of Justice, under the conditions and within the limits laid down

by the Statute, where there is a serious risk of the unity or consistency of Union law being affected.

## 7.7 Community 7: Discrimination Based on Nationality

This cluster of cases, judging from the content of the top 10 cases, deals predominantly with issues in regards to the prohibition in EU law against discrimination on grounds of nationality (TFEU art. 18) and the rights of EU citizens to freely move and reside within the territory of the member states in the EU (TFEU art. 21).

**TFEU art. 18 (ex TEC art. 12)**

Within the scope of application of the Treaties, and without prejudice to any special provisions contained therein, any discrimination on grounds of nationality shall be prohibited. The European Parliament and the Council, acting in accordance with the ordinary legislative procedure, may adopt rules designed to prohibit such discrimination.

**TFEU art. 21 (ex TEC art. 18)**

1. Every citizen of the Union shall have the right to move and reside freely within the territory of the Member States, subject to the limitations and conditions laid down in the Treaties and by the measures adopted to give them effect.

2. If action by the Union should prove necessary to attain this objective and the Treaties have not provided the necessary powers, the European Parliament and the Council, acting in accordance with the ordinary legislative procedure, may adopt provisions with a view to facilitating the exercise of the rights referred to in paragraph 1.

3. For the same purposes as those referred to in paragraph 1 and if the Treaties have not provided the necessary powers, the Council, acting in accordance with a special legislative procedure, may adopt measures concerning social security or social protection. The Council shall act unanimously after consulting the European Parliament.

## 7.8 Community 8: Restrictions on Import

This cluster of cases mostly deal with the prohibition in EU law against quantitative restrictions on imports (raised by one EU member state against another) and against all measures that have an equivalent effect (TFEU art. 34).

### TFEU art. 34 (ex TEC art. 28 )

Quantitative restrictions on imports and all measures having equivalent effect shall be prohibited between Member States.

## 7.9 Community 9: Gender Discrimination

The cases in this cluster deals with issues in regards to the prohibition against gender discrimination in regards to pay for work. According to EU law each Member State shall ensure that the principle of equal pay for male and female workers for equal work or work of equal value is applied (TFEU art. 157).

### TFEU art. 157 (ex TEC art. 141)

1. Each Member State shall ensure that the principle of equal pay for male and female workers for equal work or work of equal value is applied.

2. For the purpose of this Article, ”pay” means the ordinary basic or minimum wage or salary and any other consideration, whether in cash or in kind, which the worker receives directly or indirectly, in respect of his employment, from his employer. Equal pay without discrimination based on sex means: (a) that pay for the same work at piece rates shall be calculated on the basis of the same unit of measurement; (b) that pay for work at time rates shall be the same for the same job.

3. The European Parliament and the Council, acting in accordance with the ordinary legislative procedure, and after consulting the Economic and Social Committee, shall adopt measures to ensure the application of the principle of equal opportunities and equal treatment of men and women in matters of employment and occupation, including the principle of equal pay for equal work or work of equal value.

4. With a view to ensuring full equality in practice between men and women in working life, the principle of equal treatment shall not prevent any Member State from maintaining or adopting measures providing for specific advantages in order to make it easier for the underrepresented sex to pursue a vocational activity or to prevent or compensate for disadvantages in professional careers.

## 7.10 Community 10: Distribution of Powers

The top 10 cases in this community revolve around the distribution of powers between the European Union and the members states. This issue is regulated in TFEU art. 4 and 5. The substantive content of the cases are mostly about the clearing of accounting for agricultural subsidies emanating from EU sources and the use of fisheries quota allocated by the EU.

### TFEU Article 4

1. In accordance with Article 5, competences not conferred upon the Union in the Treaties remain with the Member States.

2. The Union shall respect the equality of Member States before the Treaties as well as their national identities, inherent in their fundamental structures, political and constitutional, inclusive of regional and local self-government. It shall respect their essential State functions, including ensuring the territorial integrity of the State, maintaining law and order and safeguarding national security. In particular, national security remains the sole responsibility of each Member State.

3. Pursuant to the principle of sincere cooperation, the Union and the Member States shall, in full mutual respect, assist each other in carrying out tasks which flow from the Treaties. The Member States shall take any appropriate measure, general or particular, to ensure fulfilment of the obligations arising out of the Treaties or resulting from the acts of the institutions of the Union. The Member States shall facilitate the achievement of the Union's tasks and refrain from any measure which could jeopardise the attainment of the Union's objectives.

**TFEU Article 5 (ex Article 5 TEC)**

1. The limits of Union competences are governed by the principle of conferral. The use of Union competences is governed by the principles of subsidiarity and proportionality.

2. Under the principle of conferral, the Union shall act only within the limits of the competences conferred upon it by the Member States in the Treaties to attain the objectives set out therein. Competences not conferred upon the Union in the Treaties remain with the Member States.

3. Under the principle of subsidiarity, in areas which do not fall within its exclusive competence, the Union shall act only if and in so far as the objectives of the proposed action cannot be sufficiently achieved by the Member States, either at central level or at regional and local level, but can rather, by reason of the scale or effects of the proposed action, be better achieved at Union level. The institutions of the Union shall apply the principle of subsidiarity as laid down in the Protocol on the application of the principles of subsidiarity and proportionality. National Parliaments ensure compliance with the principle of subsidiarity in accordance with the procedure set out in that Protocol.

4. Under the principle of proportionality, the content and form of Union action shall not exceed what is necessary to achieve the objectives of the Treaties. The institutions of the Union shall apply the principle of proportionality as laid down in the Protocol on the application of the principles of subsidiarity and proportionality.

# References


[1] L. Breiman, J. Friedman, C. J. Stone, R. Olshen, *Classification and Regression Trees* (Chapman and Hall/CRC, 1984), first edn.

[2] T. Saito, M. Rehmsmeier, *PLOS ONE* **10**, 1 (2015).

[3] M. Rosvall, D. Axelsson, C. T. Bergstrom, *Eur. Phys. J. Special Topics* **178** (2009).

[4] J. Lindholm, M. Derlén, M. Rosvall, A. Mirshahvalad, *Europaróttslig Tidskrift* **3**, 517– (2012).

[5] S. Fortunato, D. Hric, *Physics Reports* **659**, 1 (2016).
